# Interplay of Nkx3.2, Sox9 and Pax3 Regulates Chondrogenic Differentiation of Muscle Progenitor Cells

**DOI:** 10.1371/journal.pone.0039642

**Published:** 2012-07-02

**Authors:** Dana M. Cairns, Renjing Liu, Manpreet Sen, James P. Canner, Aaron Schindeler, David G. Little, Li Zeng

**Affiliations:** 1 Program in Cellular, Molecular and Developmental Biology, Sackler School of Graduate Biomedical Sciences, Tufts University, Boston, Massachusetts, United States of America; 2 Orthopaedic Research & Biotechnology Unit, The Children's Hospital at Westmead, Westmead, Australia; 3 Faculty of Medicine, University of Sydney, Sydney, Australia; 4 Building Diversity in Biomedical Research Program (BDBS), Tufts University School of Medicine, Massachusetts, United States of America; 5 Department of Anatomy and Cellular Biology, Tufts University School of Medicine, Boston, Massachusetts, United States of America; 6 Department of Orthopaedic Surgery, Tufts Medical Center, Boston, Massachusetts, United States of America; William Harvey Research Institute, Barts and The London School of Medicine and Dentistry, Queen Mary University of London, United Kingdom

## Abstract

Muscle satellite cells make up a stem cell population that is capable of differentiating into myocytes and contributing to muscle regeneration upon injury. In this work we investigate the mechanism by which these muscle progenitor cells adopt an alternative cell fate, the cartilage fate. We show that chick muscle satellite cells that normally would undergo myogenesis can be converted to express cartilage matrix proteins *in vitro* when cultured in chondrogenic medium containing TGFß3 or BMP2. In the meantime, the myogenic program is repressed, suggesting that muscle satellite cells have undergone chondrogenic differentiation. Furthermore, ectopic expression of the myogenic factor Pax3 prevents chondrogenesis in these cells, while chondrogenic factors Nkx3.2 and Sox9 act downstream of TGFß or BMP2 to promote this cell fate transition. We found that Nkx3.2 and Sox9 repress the activity of the Pax3 promoter and that Nkx3.2 acts as a transcriptional repressor in this process. Importantly, a reverse function mutant of Nkx3.2 blocks the ability of Sox9 to both inhibit myogenesis and induce chondrogenesis, suggesting that Nkx3.2 is required for Sox9 to promote chondrogenic differentiation in satellite cells. Finally, we found that in an *in vivo* mouse model of fracture healing where muscle progenitor cells were lineage-traced, Nkx3.2 and Sox9 are significantly upregulated while Pax3 is significantly downregulated in the muscle progenitor cells that give rise to chondrocytes during fracture repair. Thus our *in vitro* and *in vivo* analyses suggest that the balance of Pax3, Nkx3.2 and Sox9 may act as a molecular switch during the chondrogenic differentiation of muscle progenitor cells, which may be important for fracture healing.

## Introduction

Satellite cells are the tissue specific stem cells in the adult skeletal muscle. These cells lie beneath the basement membrane of the muscle fiber and are usually mitotically quiescent [1]. Upon injury or when challenged with a variety of mechanical or biochemical stimuli, satellite cells re-enter the cell cycle and give rise to differentiated myocytes, which form new muscle fibers or fuse with existing fibers, and contribute to muscle growth and repair [1]. Satellite cells from the trunk and the limb are derived from an embryonic population of progenitor cells in the somites, transient mesodermal structures that develop on either side of the neural tube [1]. These embryonic progenitor cells are characterized by the expression of transcription factors Pax3 and Pax7, which are important for muscle differentiation and survival [2] and for specifying the muscle satellite cell population responsible for postnatal growth [1], [3]. Upon activation, satellite cells rapidly initiate MyoD expression, which leads to the activation of myogenin, and terminally differentiated structural muscle genes such as myosin heavy chain (MHC) [1], [3]. Interestingly, recent data indicated that although MyoD is not expressed in quiescent satellite cells in the adult, it is transiently expressed in satellite cell progenitors in the embryo, suggesting that satellite cells are derived from committed embryonic precursors of myogenic lineage [4], [5].

Initially, satellite cells were considered to be unipotent stem cells with the ability of generating a unique specialized phenotype, the skeletal muscle cells. However, satellite cells have since been shown to have the ability to adopt alternative cell fates. One such alternative cell fate is the adipogenic fate, as Pax7(+) satellite cells isolated from single myofibers adopted adipogenic fate, in addition to muscle fate *in vitro* [6], [7]. Another alternative cell fate is the osteogenic fate, as muscle satellite cells have been shown to be induced by BMPs to differentiate into osteoblasts in culture [7], [8], [9], [10].

Satellite cells have also been shown to have the capacity to form cartilage. *In vivo*, Pax7(+) satellite cells were found to contribute to cartilage growth in salamanders during limb regeneration after amputation [7], [11]. Furthermore, lineage-labeled satellite cells were found to express cartilage marker collagen II in a mouse model of fracture healing [12]–[13]. Such satellite cells accumulate in the callus tissue of the fracture site, exhibit the typical morphology of chondrocytes and participate in cartilage formation, which is an essential step in fracture healing [14], [15]. In fact, when a physical barrier (a cell impermeable membrane) was introduced between the muscle and fractured bone, subsequent fracture healing was significantly impaired [16]. Consistently, when isolated muscle was infected with BMP2, it served as a superior bridge for fracture repair [17]. *In vitro*, L6 myoblasts and C2C12 myoblasts were reported to differentiate into chondrocytes when treated with demineralized bone matrix or BMP2 [18], [19], [20], [21]. These observations suggest that muscle satellite cells or myoblasts can undergo chondrogenic differentiation, and that this process may play an important role in cartilage and regeneration during fracture healing. However, the molecular mechanisms by which muscle satellite cells adopt a cartilage fate still remain elusive. While TGFß/BMP signaling has been shown to be important in this process, very little is known about how downstream intracellular factors regulate cell fate transition in muscle progenitor cells.

In this work, we have characterized the molecular events that lead to the adoption of cartilage cell fate in muscle satellite cells. We demonstrate that two transcription factors, Nkx3.2 and Sox9, act downstream of TGFß/BMP signaling to regulate the transition from myogenic fate to a chondrogenic fate. Nkx3.2 and Sox9 were both found to promote chondrogenesis in the satellite cells, but Nkx3.2 strongly inhibits the adoption of muscle cell fate while Sox9 only weakly inhibits myogenesis in satellite cells. A reverse function mutant of Nkx3.2 blocks the activity of Sox9, suggesting that Nkx3.2 is required for Sox9 to promote cartilage formation in satellite cells. In addition, we found that the muscle-determining factor Pax3 strongly inhibits chondrogenesis. To explore the *in vivo* significance of these factors, we used a mouse fracture healing model in a genetically modified reporter mouse where muscle progenitors were lineage-traced. We found that in the descendents of muscle progenitors that contributed to cartilage formation, Nkx3.2 and Sox9 were strongly induced, while Pax3 expression was strongly repressed. Together, our data suggest that the balance of Nkx3.2, Sox9 and Pax3 can act as a molecular switch during the chondrogenic differentiation of satellite cells, which may play an important role in the healing process *in vivo*.

## Materials and Methods

### Satellite Cell Isolation

Chicken eggs were purchased from Hy-line Inc., Pennsylvania. Satellite cells were isolated from day 17 chicken pre-hatch embryos according to an established protocol [22]. Briefly, pectoral muscles were dissected and placed into sterile PBS with penicillin/streptomycin and then minced. Ground muscle was placed in a centrifuge tube and digested with pronase (1 mg/ml in PBS) in a 37°C water bath for 40 min, with agitation every 10 min. The tube was centrifuged for 4 min at 3000 rpm. The supernatant was then discarded, and the pellet was resuspended with PBS and vortexed briefly. The tube was then centrifuged for 10 min at 1000 rpm three times, and the satellite cell-containing supernatants from each cycle were saved and pooled into a new sterile 50 ml centrifuge tube. The supernatants were then passed through a 40 µM cell strainer (BD Biosciences, San Jose, CA) to obtain a single cell suspension, centrifuged for 6 minutes at 3000 rpm and the resulting supernatants were discarded. The cell pellet was resuspended in medium: DMEM (Invitrogen, CA), 10% FBS (Hyclone, IL) and 1% penicillin/streptomycin (Invitrogen); and then cells were plated on tissue culture plates. Plates were incubated for 24 hours in a humidified 37°C, 5% CO_2_ incubator, and then washed with sterile PBS to remove non-adherent cells. All freshly isolated cells were confirmed to be positive for satellite cell specific markers, Pax3 and Pax7, before subsequent experiments were conducted.

### Cell Culture

Satellite cells were cultured in DMEM with 10% FBS (Hyclone, Logan, UT) and 1% antibiotic/mycotic (Invitrogen, CA). For chondrogenic induction, satellite cells were plated as high density micromass cultures in the presence of chondrogenic media: DMEM (Invitrogen), supplemented with ITS (insulin, transferrin, selenate, Sigma Cat#12521), 0.1 mM ascorbic acid (Sigma, MO), human serum albumin (HSA, Sigma), 10^−7^ M dexamethasone, and 10 ng/ml TGFß3 (R&D, MN) or BMP2 (R&D)) [23]–[24]. Briefly, cells were split and resuspended as 10^5^ cells/10 µl droplet, and pipetted onto a plate and allowed to adhere in a 37°C, 5% CO_2_ incubator for approximately one hour before the addition of chondrogenic media. All cells were grown for 5 days before they were subject to histological and qRT-PCR analysis.

### Virus production and infection of satellite cells

Avian-specific retroviruses (RCAS) were generated by first transfecting chick embryonic fibroblasts (CEF) with retroviral constructs encoding for the following genes: GFP, Nkx3.2HA, Sox9V5, Alkaline phosphatase (AP), Pax3HA, Nkx3.2ΔC-HA (deletion of C-terminus from aa219–278), or Nkx3.2ΔC-VP16 [25], [26], [27]. The viral supernatant was concentrated by ultracentrifugation (21,000 rpm, 2 hrs), and titered by directly visualizing GFP expression (in the case of RCAS-GFP) or indirect immunocytochemistry using anti-GAG antibody (which recognizes the viral coat protein GAG). Viruses with titers of at least 10^8^ particles/ml were used in all satellite cell cultures. For co-infection experiments, viruses of different coat proteins A- or B- were used. Retroviral infection of satellite cells was carried out by directly adding concentrated virus into growing cell cultures. High levels of expression were detectable 48 hrs post-infection, at which time the cells were split and used in subsequent experiments.

### Luciferase Assays

For the construction of the luciferase construct, a published murine Pax3 promoter sequence (1.5 kb) [28] was cloned into SmaI and NheI sites of the pGL3 luciferase vector (Promega, Wisconsin). Satellite cells were transfected with pGL3-Pax3 promoter construct or pGL3 control using Fugene6 according to the manufacturer's protocol. After 48 hrs, cells were processed using the Luciferase Assay System (Promega, Wisconsin). Briefly, cells were thoroughly lysed with lysis buffer using a freeze-thaw cycle. Supernatants were added to the luciferase assay reagent in a 96 well plate, then immediately read on a 1450 Microbeta Wallac Trilux plate reading luminescence counter (Perkin Elmer, MA). At least three independent experiments were performed to obtain reproducible results.

### Histological analyses

All samples were fixed with 4% paraformaldehyde (Sigma). For alcian blue staining, cryosections of satellite cell micromass cultures were pre-washed with 0.1 N HCl then incubated with 1% (w/v) alcian blue (Sigma) overnight, followed by repeated washes with 0.1 N HCl. Hematoxylin and eosin (H&E) (Sigma) staining was carried out according to standard protocol on cryosectioned mouse tissues. Staining for heat-inactivated alkaline phosphatase (HI-AP) on serial cryosectioned mouse tissue was achieved by incubating the slides at 75°C for 50 min, to eliminate endogenous AP activity, followed by application of NBT (100 mg/ml in 70% dimethyl formamide) and BCIP (50 mg/ml in dimethyl formamide) (Invitrogen, CA).

For immunocytochemistry, the following primary antibodies were used: mouse anti-Collagen II (generous gift from Dr. Tom Linsenmayer, Tufts University), rabbit anti-Collagen II (Abcam, MA), rabbit anti-Sox9 (Chemicon, MA), mouse anti-Pax3 (Developmental Studies Hybridoma Bank (DSHB), IA), mouse anti-Pax7 (DSHB), mouse anti-Myosin Heavy Chain (MHC) (MF20 from DSHB), rabbit anti-HA (Sigma), rabbit anti-V5 (Sigma), rabbit anti-VP16 (Abcam), mouse anti-CD34 (AbD Serotec), mouse anti-CD45 (AbD Serotec), mouse anti-M-cadherin (BD Transduction Laboratories), rabbit anti-Myf5 (Santa Cruz), rabbit anti-Desmin (Abcam), and rat anti-Sca-1 (AbD Serotec). For immunohistochemistry of mouse tissues, cryosections were first subject to antigen retrieval by treating slides with 1% sodium dodecyl sulfate (SDS) in phosphate-buffered saline (PBS) for 5 min at room temperature prior to subsequent staining steps. For all other immunocytochemistry of cell culture, no antigen retrieval was used. All samples were first blocked with PBS with 0.1% Triton X-100 (Sigma) and 6% goat serum (Sigma), then incubated with primary antibodies overnight. After repeated washes with PBS with 0.1% Tween (PBST), cultures were incubated with secondary antibodies. For immunofluorescent staining, all secondary antibodies used were conjugated with Alexa 488 (green) or 594 (red) (Invitrogen, CA), and all cultures were counterstained with DAPI (Invitrogen, CA). For colorimetric immunostaining, the secondary antibody (Vector Laboratories, CA) was conjugated with biotin, and the signal was amplified using the Vectastain Elite ABC kit (Vector Laboratories, CA) and developed using DAB-peroxidase (Sigma).

### Reverse Transcriptase-Polymerase Chain Reaction (RT-PCR) Analysis

RNA was isolated from all cell cultures using the RNeasy mini-kit from Qiagen (Chatsworth, CA). For RNA samples isolated from mouse tissue cryosections using laser capture microscopy (LCM), Qiagen MicroKit was used (Chatsworth, CA). cDNA was generated using MLV-reverse transcriptase (Invitrogen, CA) according to standard protocol. All quantitative PCRs were performed on the iQ5 Real-Time PCR Detection System (BioRad, Hercules, CA). At least three independent experiments were performed to obtain reproducible results.

All PCR analyses of *in vitro* experiments were normalized to GAPDH. All PCR analyses from *in vivo* mouse LCM samples were normalized to the 18S RNA. Sequences for all primers are listed in “Supporting information”, Table S1. All PCR primers were designed to amplify 100–200 bp of each gene for compliance with the requirement of the real time PCR machine.

### Western Blot analysis

For Western Blot analysis, total protein lysates were obtained following a standard protocol from confluent 6 cm tissue culture plates containing roughly 3×10^6^ cells [25]. The proteins were separated by SDS-PAGE using BioRad mini-gel apparatus and blotted onto nitrocellulose membranes using BioRad transfer apparatus. The membranes were blotted with the following antibodies overnight: rabbit anti-Collagen II (Abcam) and mouse anti-ß-actin (Abcam). After repeated washing, the membranes were hybridized with secondary antibodies of goat anti-mouse or goat anti-rabbit HRP conjugated antibodies (Calbiochem). The signals were developed using Pierce ECL substrate (cat# 32106), and Kodak films exposed to chemiluminescent signals were developed in Kodak M35A X-OMAT processor.

### Glycosaminoglycan (GAG) assay

Virus-infected muscle satellite cells were seeded into silk porous scaffold discs (5 mm in diameter, 3 mm in height, pore size: 500–600 µm, a generous gift from Dr. David Kaplan, Tufts University) at 10^5^ cells/scaffold [29] and cultured for 7 days. Silk scaffolds were digested overnight using a standard papain digestion cocktail (125 µg/mL papain, 5 mM L-cysteine, 100 mM Na2HPO4, and 5 mM EDTA, pH 6.2) at 60°C. For total glycosaminoglycan (GAG) analysis, 1,9-dimethylmethylene blue (DMMB) assay was used [30]. Briefly, the papain-digested samples were mixed with DMMB reagent and absorbance was measured at 525 nm. GAG was estimated using a standard curve generated using shark chondroitin sulfate (Sigma).

### Culturing chondrogenic cells in the living bone

To create the culture environment for long bone growth, fertilized chicken eggs were incubated for 3 days, at which time a small window was created in the egg shell to expose the chorioallantoic membrane (CAM), which is rich in blood supply [31], [32], [33]. Mouse humeri along with the surrounding muscle and connective tissues were isolated from postnatal day 7 mice (C57B6 strain). Virus-infected muscle satellite cells were seeded into silk porous scaffold discs (2 mm in diameter, 2 mm in height, pore size: 500–600 µm, a generous gift from Dr. David Kaplan) at 10^4^ cells/scaffold [29]. To implant the satellite cell constructs, the skin and fascia were first carefully retracted to expose the underlying bone, and an incision was made. The cell/scaffold construct was then placed between the bone segments, and secured in place using a 30G syringe needle. The skin and fascia were then pulled back over the resected bone, and the entire limb was placed on top of the CAM. Finally, the window of the egg shell was sealed with plastic tape, and the egg was placed in a humidified 37°C incubator for 7 days before histological analysis.

### Microscopy

Bright-field and fluorescent images from histological and immunocytochemistry analyses were taken under the Olympus IX71 inverted microscope using Olympus DP70 digital camera and associated software (Olympus, PA). Laser capture microscopy (LCM) was performed using the Arcturus PixCell IIe system (Tufts Imaging Facility, Center for Neuroscience Research) using the established protocol [34], [35]. Briefly, cryosectioned tissues were dehydrated and were overlaid with a thermoplastic membrane, which was mounted on an optically transparent cap (Arcturus Macro LCM caps, Applied Biosystem, CA). Target tissues were identified by comparisons with serial sections that were stained with heat-inactivated alkaline phosphatase (HI-AP). Upon laser activation, target cells were captured by focal melting of the membrane, then the captured tissue was immersed in a denaturation solution and was subsequently subject to RNA isolation.

### Fracture creation in MyoD-cre Z/AP labeled mice

MyoD-cre Z/AP reporter mice were bred by the crossing of the MyoD-cre [4] and Z/AP [36] lines. The *MyoD*-Cre mouse line was a gift from Dr. David Goldhamer (University of Connecticut, Storrs, USA). The Z/AP line was supplied by Prof. Patrick Tam (Children's Medical Research Institute, Westmead, NSW, Australia) with permission from Prof. Andras Nagy (Samuel Lunenfeld Research Institute, Toronto, Ontario, Canada). The cross strain labels all MyoD(+) lineage cells to permanently express the heat-resistant human placental alkaline phosphatase (hPLAP). Midshaft tibial fractures were generated in anaesthetized 2–3 month old MyoD-cre Z/AP mice and littermate controls by manual three-point using a previously published model [13], [37]. Tissue specimens harvested from mice of 1 week post fracture were used for enzymatic and immunohistochemical staining, as well as laser capture microscopy (LCM) studies. Animal experimentation was approved by the CHW/CMRI Animal Ethics Committee (K248) and the Westmead Hospital Animal Ethics Committee (4102).

### Statistical Analysis

For statistical analysis, the mean and standard deviation were calculated and are shown in all figures. At least three independent experiments were performed to obtain reproducible results, and at least three independent samples or slides were analyzed in each experiment. Statistically significant differences (i.e., p<0.05) were determined by one-factor analysis of variance (ANOVA) with post hoc Tukey test using the statistics software SYSTAT12 (Systat, Chicago, IL, USA). Conclusions on alteration of gene expression (such as upregulation and downregulation) were drawn based on statistical analysis.

## Results

### Isolated muscle satellite cells can be differentiated into chondrocytes at the expense of normal muscle cell differentiation

**Figure 1:**
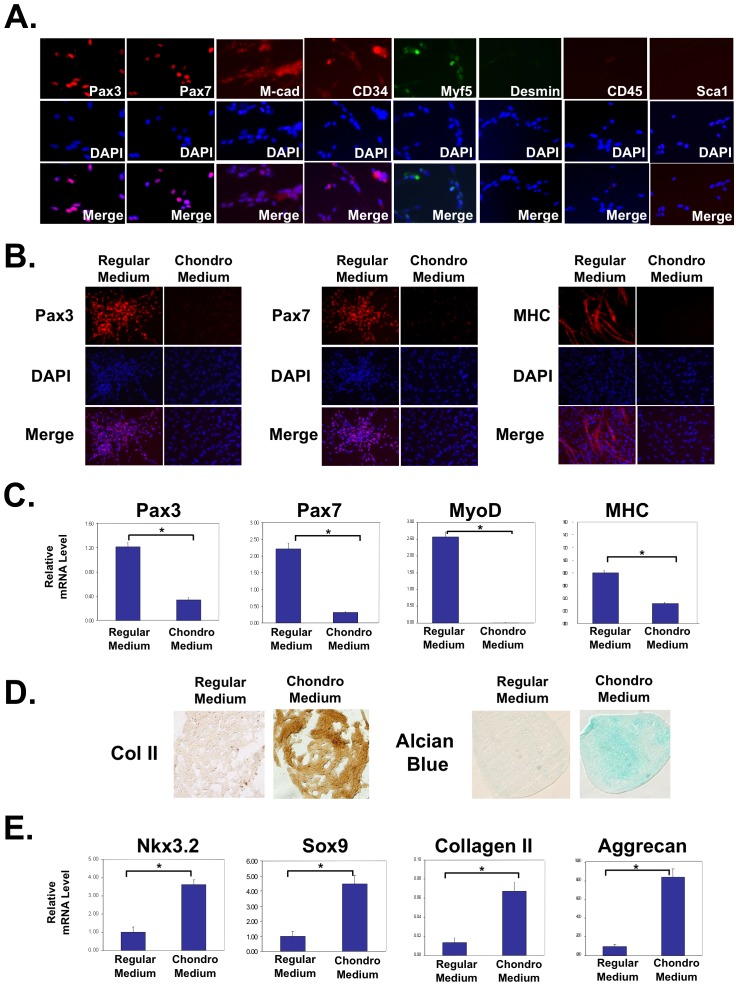
Isolated muscle satellite cells can be redirected toward a cartilage phenotype at the expense of the default muscle phenotype. (A) Immunocytochemistry analysis indicating that isolated chicken muscle progenitor cells at day 0 are over 95% positive for Pax3, Pax7, M-cadherin and CD34 (red staining). They are 33%-positive for Myf5 and are negative for Desmin, CD45 and Sca-1. Percentage positive cells = number of positively stained cells/total cell number (based on DAPI staining). (B) Immunocytochemistry results indicating a dramatic downregulation of Pax3, Pax7, and MHC in response to chondrogenic stimuli. (C) qRT-PCR analysis showing decreased expression of muscle markers Pax3, Pax7, MyoD, and MHC in chondrogenic media as compared to those cultured in regular media. (D) Immunocytochemistry analysis on sectioned micromass cultures showing increased collagen II protein expression upon chondrogenic media treatment. Alcian Blue staining indicates increased glycosaminoglycan levels when cultured in chondrogenic media as compared to those cultured in regular media. (E) qRT-PCR analysis showing increased expression of cartilage markers Nkx3.2, Sox9, collagen II, and aggrecan in chondrogenic media as compared to those cultured in regular media. All PCR results were normalized to GAPDH. “*” denotes p<0.05 in statistical analysis.

For chondrogenic differentiation of myogenic cells, muscle satellite cells were grown as micromass cultures in chondrogenic medium. Satellite cells were isolated from the pectoralis muscles of late stage chicken embryos, which give rise to muscle cells that are phenotypically similar to those from adult muscle [38]. The identity of muscle satellite cells was confirmed by immunocytochemistry analysis, which indicated that our cells were ≥95% positive for satellite cells markers Pax3, Pax7, M-cadherin and CD34 at day 0 (D0) (Figure 1A) [39], [40]. Some cells (33%) were positive for Myf5 (Figure 1A), suggesting that some of the satellite cells had become activated during the isolation [2]. As none of these cells were positive for Desmin (Figure 1A) or myosin heavy chain (MHC) (data not shown), it indicates that the muscle satellite cells had not undergone myogenic differentiation [41]. We further confirmed the identity of the satellite cells by evaluating the expression of other lineage markers. We found that these satellite cells did not express CD45 or Sca-1, thus we believe that they did not contain hematopoietic stem cells or fibrocyte progenitors [39], [40]. Quantification of cell lineage markers is shown in supplemental information (Figure S1). Furthermore, when these cells were differentiated, nearly 100% of them were positive for myogenic differentiation marker Desmin (Figure S1). We then cultured the satellite cells in three-dimensional (3D) micromass cultures in the presence of the standard chondrogenic media containing TGFß3 [23], [24]. This culture system has been established to differentiate embryonic progenitor cells or bone marrow-derived mesenchymal stem cells into cartilage cells [23], [24], [42]. Indeed, our immunocytochemistry and RT-PCR analyses indicated that upon treatment with chondrogenic medium for 5 days, there was a dramatic reduction of Pax3 and Pax7, as well as committed myoblast marker MyoD, and differentiated myocyte marker myosin heavy chain (MHC) (Figure 1C and 1D). Concurrently, culturing with chondrogenic medium resulted in the induction of cartilage matrix, as shown by immunocytochemistry analysis of cartilage marker collagen II (Figure 1E), as well as alcian blue staining which reflects glycosaminoglycan (GAG) content (Figure 1E) [43]. To determine the efficiency of chondrogenesis, we dissociated the condensed cells from micromass cultures and evaluated collagen II expression by immunocytochemistry. We found that almost all cells treated with the chondrogenic medium exhibited significantly higher levels of collagen II (Figure S2). This was further confirmed by qRT-PCR analysis, which showed that chondrogenic medium treatment led to the induction of transcription factors Nkx3.2 and Sox9, as well as cartilage matrix markers collagen II and aggrecan (Figure 1F). Replacing TGFß with BMP2 in the chondrogenic medium led to similar results (data not shown). We found that satellite cells of a later passage (P2) had a reduced capacity for chondrogenic differentiation (Figure S3), therefore we opted to use passage 0 satellite cells for future experiments. Furthermore, in our time course study, we found that while myogenic gene expression (Pax3, Pax7 and MyoD) showed a marked decrease at day 5 and continued into day 12 in cells cultured in chondrogenic medium, chondrocyte markers Nkx3.2, Sox9 and aggrecan were significantly increased in expression at day 5 (Figure S4). This suggests that the transition from the myogenic fate to chondrogenic fate may have begun at day 5, which is the time point we opted to use for subsequent experiments. Taken together, our data clearly demonstrate that muscle satellite cells can be driven to adopt a cartilage phenotype *in vitro* at the expense of the default muscle cell fate.

### Pax3 inhibits the adoption of cartilage cell fate by muscle satellite cells

**Figure 2:**
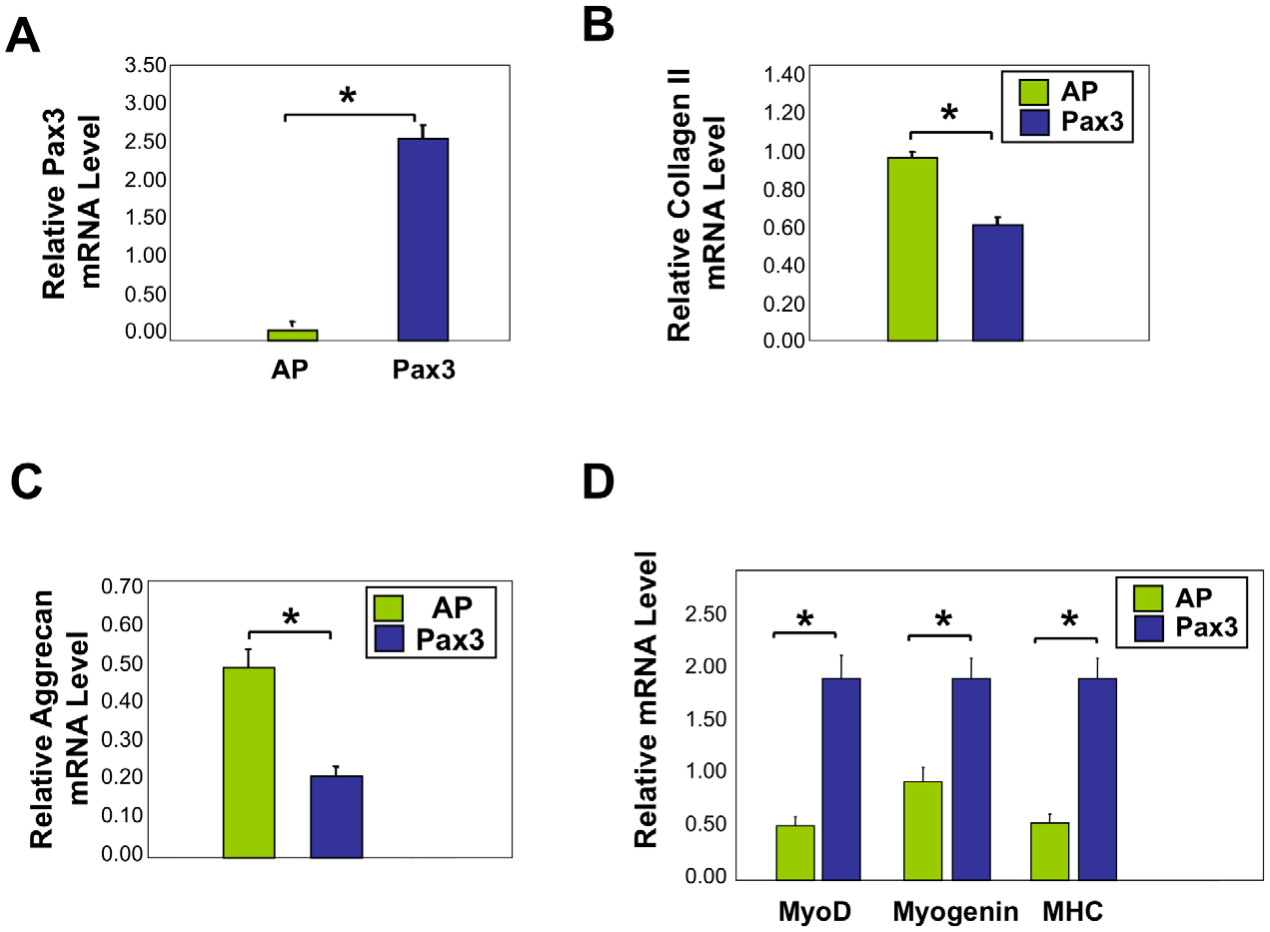
Viral infection with Pax3 in muscle satellite cells inhibits chondrogenesis while maintaining muscle gene expression. qRT-PCR analysis of satellite cells infected with RCAS-A-Pax3 or RCAS-A-AP (alkaline phosphatase, control virus) and cultured in 3D micromasses in chondrogenic media. (A) Pax3 mRNA, (B) Collagen II mRNA, (C) Aggrecan mRNA, and (D) MyoD, Myogenin, MHC mRNA expression. All PCR results were normalized to GAPDH. “*” denotes p<0.05 in statistical analysis.

We next investigated the roles of intracellular factors on the chondrogenic differentiation of satellite cells. Upon chondrogenic differentiation of muscle satellite cells, muscle fate determining factor Pax3 expression was strongly downregulated (Figure 1C and 1D). Therefore, we hypothesized that Pax3 would negatively regulate the differentiation of satellite cells to chondrocytes. To test this hypothesis, we infected muscle satellite cells with a Pax3HA-encoding retrovirus, and cultured these cells in 3D micromass in chondrogenic medium. We confirmed the ectopic expression of Pax3 by qRT-PCR analysis (Figure 2A). Significantly, while control virus-infected cells maintained a spherical appearance in the course of micromass cultures, Pax3-infected cells showed a much more elongated appearance (Figure S5), although we could not clearly distinguish the differences in cell shape due to cell condensation that accompanies chondrogenesis. At the gene expression level, forced expression of Pax3 in chondrogenic medium caused a significant decrease in expression of cartilage markers collagen II and aggrecan as compared to controls (Figure 2B and 2C), while leading to an increase in the expression of muscle markers MyoD, myogenin, and MHC (Figure 2D). We did not observe any differences in the expression levels of PPARγ, osteopontin or vimentin, suggesting that Pax3 may not have promoted adipocyte, fibrocyte or bone lineages in the satellite cells (Figure S6) [39], [40]. Taken together, our results indicate that Pax3 inhibits chondrogenesis in muscle satellite cells, and suggest that for muscle satellite cells to differentiate into chondrocytes, Pax3 expression must first be inhibited.

### Nkx3.2 and Sox9 inhibit muscle gene expression in muscle satellite cells

**Figure 3:**
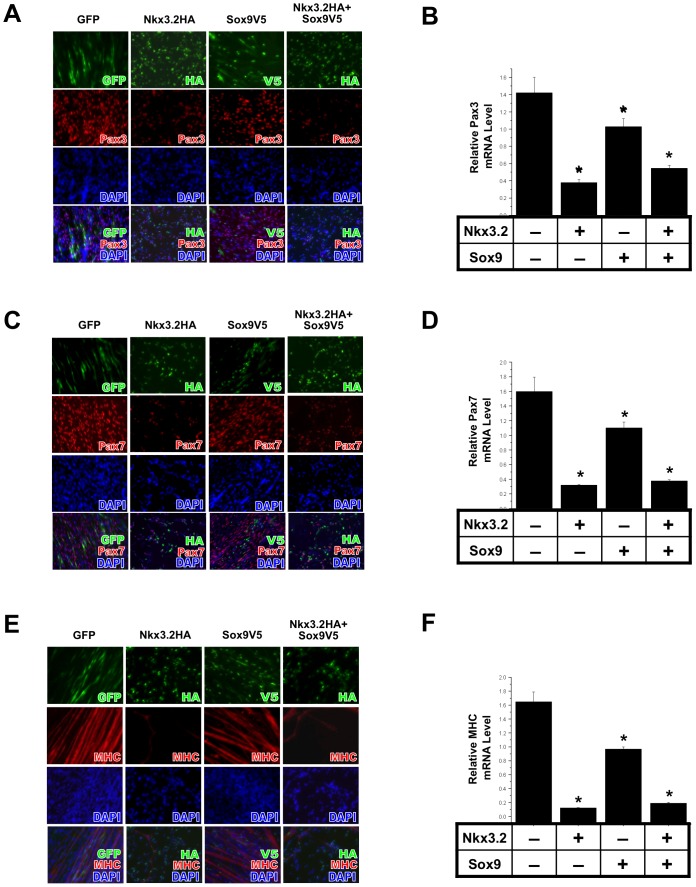
Nkx3.2 and Sox9 inhibit muscle-specific gene expression in muscle satellite cells. Muscle satellite cells were infected with RCAS viruses encoding GFP, Nkx3.2HA, Sox9V5 or Nkx3.2HA+Sox9V5. Immunocytochemistry and qRT-PCR analyses were performed. Virus staining images were overlaid with Pax3, Pax7 and MHC images, and DAPI stains cell nuclei. For all qRT-PCR, GAPDH was used for normalization. (A) Pax3 immunostaining results. (B) Pax3 mRNA expression. (C) Pax7 immunostaining results. (D) Pax7 mRNA expression. (E) MHC immunostaining results. (F) MHC mRNA expression. “*” denotes statistically significant differences (p<0.05) relative to control samples.

Next we assessed whether factors induced by chondrogenic media were capable of inhibiting the default muscle fate of satellite cells. Sox9 and Nkx3.2 are two factors induced by TGFß-containing chondrogenic medium (Figure 1F), and have been shown to promote cartilage formation during embryogenesis [44], [45], [46], [47], [48], [49]. However, it has not been determined whether these factors might influence chondrogenic differentiation of muscle satellite cells. Thus, we ectopically expressed retroviruses encoding Nkx3.2HA and/or Sox9V5, whose expression levels were confirmed by qRT-PCR (Figure S7). We found that forced expression of Nkx3.2 encoding retrovirus in muscle satellite cells strongly inhibited Pax3 expression, as Nkx3.2-expressing cells expressed less Pax3 as confirmed by immunostaining (Figure 3A) and qRT-PCR analysis (Figure 3B). Sox9, on the other hand, only weakly inhibited Pax3 expression, suggesting that Nkx3.2 is a more potent inhibitor of muscle cell fate in satellite cells (Figure 3A and 3B). Similarly, Nkx3.2 inhibited the expression of satellite cell marker Pax7 as well as MHC, a marker for differentiated myocytes (Figure 3C–3F). As these satellite cells were already expressing a high level of Pax3 and Pax7 upon isolation, the inhibition of Pax3 and Pax7 at the protein level was not as dramatic as that of MHC (Figure 3). While the effect of Sox9 on muscle gene expression was not clearly visible in the less quantitative analysis of immunocytochemistry (Figure 3A, 3C and 3E), RT-PCR analysis indicates that Sox9 consistently exhibited a significant though weak inhibition of Pax3, Pax7 and MHC expression relative to the inhibitory effect of Nkx3.2 on muscle marker expression (Figure 3B, 3D and 3F). Combinatorial treatment of Nkx3.2 and Sox9 led to inhibition of muscle cell fate similar to that of Nkx3.2 alone, suggesting that there is no synergy between these two factors and that they may not act independently in this process (Figure 3). Together, these data demonstrate that Nkx3.2, and to a much lesser extent, Sox9, are cartilage-associated transcription factors that can prevent satellite cells from adopting their default muscle fate.

### The C-terminus of Nkx3.2 is required for the inhibition of muscle cell fate in muscle satellite cells

**Figure 4:**
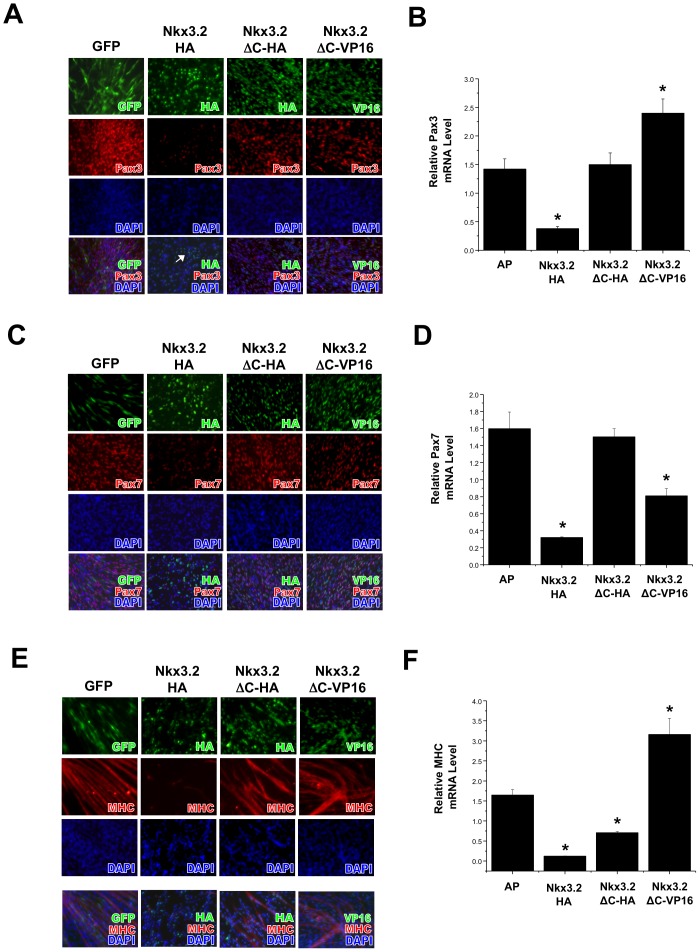
The C-terminus of Nkx3.2 is required to inhibit muscle cell fate in muscle satellite cells. Muscle satellite cells were infected with RCAS viruses encoding GFP, Nkx3.2HA, Nkx3.2-ΔCHA or Nkx3.2-ΔC-VP16. Immunocytochemistry and qRT-PCR analyses were performed. Virus staining images were overlaid with Pax3, Pax7 and MHC images, and DAPI stains cell nuclei. For all qRT-PCR, GAPDH was used for normalization. (A) Pax3 immunostaining results. (B) Pax3 mRNA expression. (C) Pax7 immunostaining results. (D) Pax7 mRNA expression. (E) MHC immunostaining results. (F) MHC mRNA expression. “*” denotes statistically significant differences (p<0.05) relative to control samples.

Given that Nkx3.2 strongly inhibited the muscle fate in satellite cells, we next determined if this effect was specific by using two Nkx3.2 mutants. In the Nkx3.2-ΔC mutant, 58 amino acids from the C-terminus were deleted. During somite differentiation in the embryo, Nkx3.2 was found to behave as a transcriptional repressor and the C-terminus was determined to be transcriptional repression domain for promoting cartilage formation [49] [26], [50]. However, it is not clear whether this domain is also required for inhibiting muscle cell fate in satellite cells. The other mutant we used was a reverse function mutant of Nkx3.2 whose C-terminus domain had been replaced by a VP16 constitutive activation domain (Nkx3.2ΔC-VP16). We found that while wild type Nkx3.2 infection led to a significantly reduced Pax3, Pax7 and MHC expression in satellite cells, Nkx3.2-C-terminus deletion mutant (Nkx3.2-ΔC-HA) did not inhibit the expression of Pax3 at the protein or mRNA level (Figure 4A and 4B). Significantly, Nkx3.2ΔC-VP16 induced the expression of Pax3 (Figure 4B). Nkx3.2 fusion with the VP16 activation domain led to the opposite phenotype as wild type Nkx3.2, suggesting that Nkx3.2 acts as a transcriptional repressor in inhibiting Pax3 expression. Similar to the effect on Pax3 expression, Nkx3.2-ΔC mutant did not inhibit Pax7 expression (Fig. 4C and 4D), suggesting that the C-terminus of Nkx3.2 is required for Pax7 repression as well. Interestingly, deletion of the C-terminus of Nkx3.2 alleviated, but did not completely abolish Nkx3.2-mediated MHC repression (Figure 4E and 4F), suggesting Nkx3.2 may inhibit MHC expression through additional domains other than the C-terminus. While replacing the C-terminus with a VP16 activation domain led to significantly enhanced expression of Pax3 and MHC, it did not lead to increased Pax7 expression in the satellite cells (Figure 4B, 4D and 4F). The effect of Nkx3.2ΔC-VP16 on Pax7 expression could be due to the intricate interaction of Pax7 with other myogenic factors, such as Pax3 and myogenin, which were both shown to inhibit Pax7 expression [51], [52].

### Nkx3.2 inhibits Pax3 promoter activity

**Figure 5:**
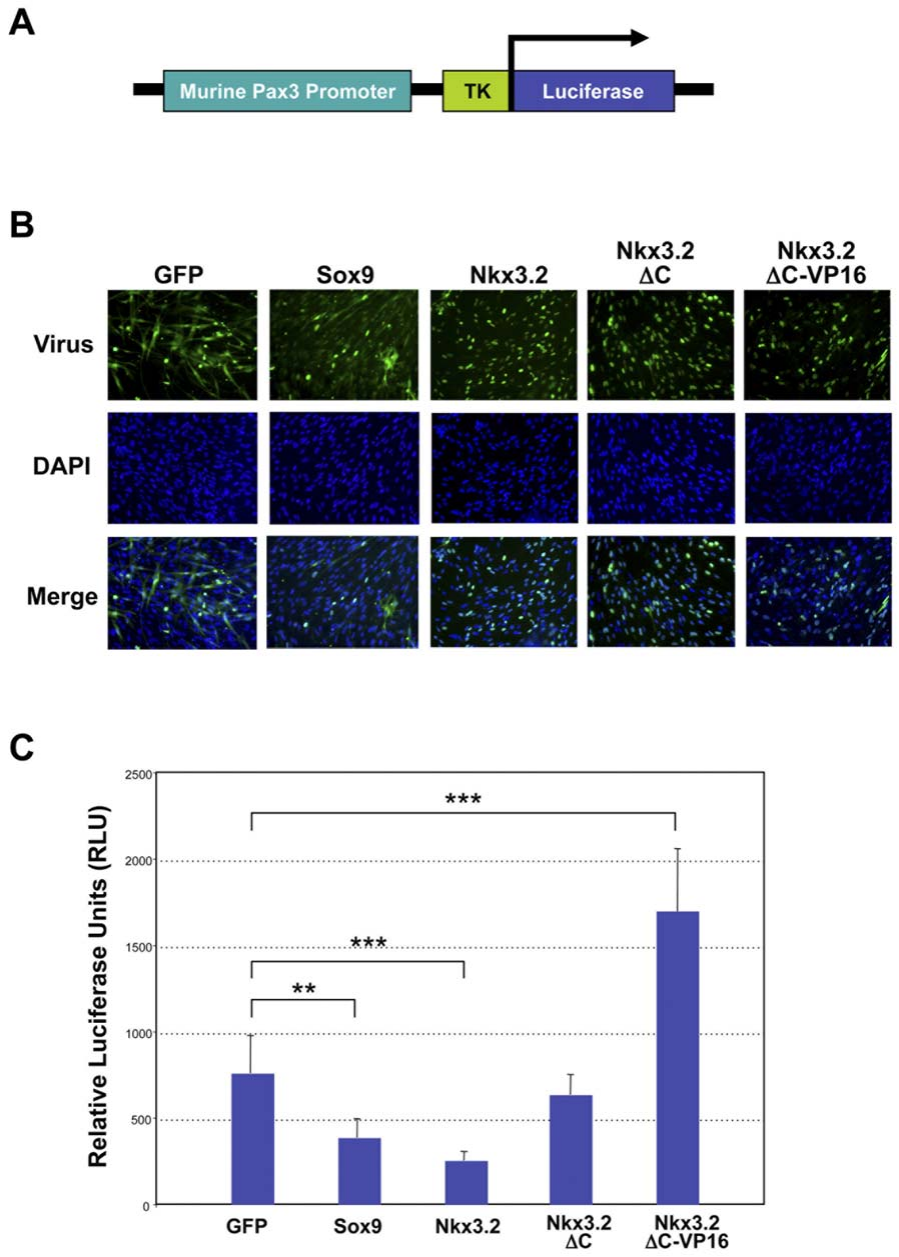
Nkx3.2 and Sox9 inhibit mouse Pax3 promoter activity. (A) Schematic diagram of the mouse Pax3 promoter luciferase construct. (B) Immunocytochemistry analysis showing equal infection efficiencies of all viruses (GFP, Sox9, Nkx3.2, Nkx3.2-ΔC-HA, Nkx3.2-ΔC-VP16). (C) Luciferase analysis on satellite cells infected with all viruses (GFP, Sox9, Nkx3.2, Nkx3.2-ΔC-HA, Nkx3.2-ΔC-VP16) and transfected with the Pax3 luciferase construct. A control luciferase vector was used for normalization. “**” denotes p<0.01 and “***” denotes p<0.001 in statistical analysis.

Since Nkx3.2 acts as a repressor to strongly inhibit Pax3 expression, we hypothesized that Nkx3.2 may inhibit Pax3 at the promoter level. A mouse Pax3 promoter sequence was previously identified from LacZ reporter analysis in transgenic mice which indicated that this promoter recapitulates endogenous Pax3 expression in the trunk [28]. Thus, we generated a luciferase reporter that contains this murine Pax3 promoter sequence to investigate whether Nkx3.2 acts directly on the Pax3 promoter to inhibit its expression (Figure 5A). For comparison, we also evaluated the effect of GFP and Sox9 on the Pax3 promoter. We first infected satellite cells with retroviruses that express GFP, Sox9V5, Nkx3.2-HA, Nkx3.2ΔC-HA, or Nkx3.2ΔC-VP16, and confirmed the efficiency of viral infection by performing immunocytochemistry (Figure 5B). We then transfected the Pax3 promoter luciferase construct into these satellite cells. We found that while Sox9 showed a moderate but significant reduction in Pax3 promoter activity, Nkx3.2 dramatically inhibited Pax3 promoter activity (Figure 5C). C-terminal deletion mutant of Nkx3.2 (Nkx3.2-ΔC) showed essentially no effect on the Pax3 promoter as compared to control GFP-infected cells (Figure 5B). Intriguingly, the Nkx3.2 reverse function mutant (Nkx3.2-ΔC-VP16) activated the Pax3 promoter by two folds (Figure 5C). Although it is unclear at this moment whether Sox9 and Nkx3.2 exert their effect on the Pax3 promoter through direct binding, our promoter study results are consistent with our analyses of endogenous Pax3 mRNA and protein expression in satellite cells, and lead us to conclude that Nkx3.2 and Sox9 may inhibit muscle gene expression by inhibiting the Pax3 promoter.

### Treatment with either Nkx3.2 or Sox9 can induce cartilage gene expression in muscle satellite cells

**Figure 6:**
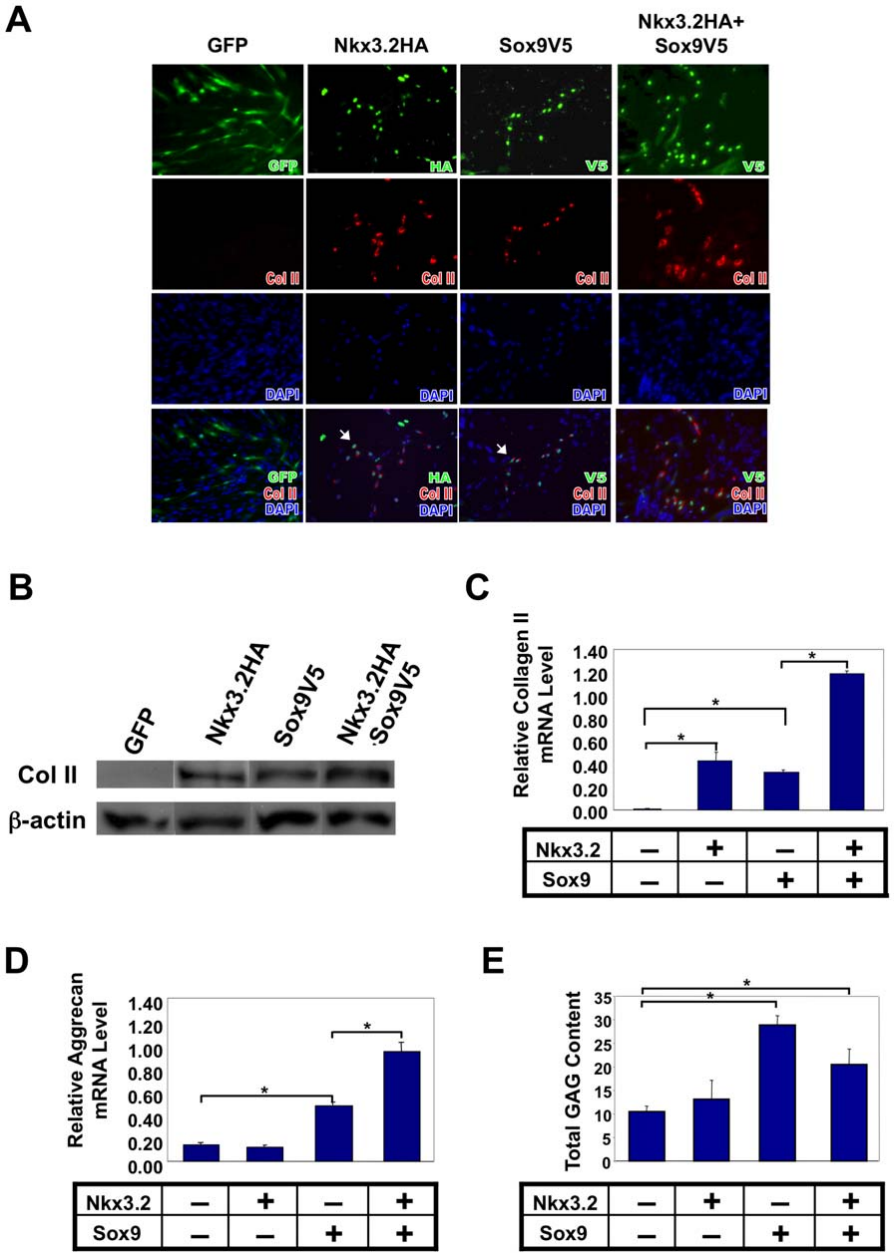
Nkx3.2 and Sox9 can induce muscle satellite cells to express cartilage markers collagen II and aggrecan. Muscle satellite cells were infected with RCAS viruses encoding GFP, Nkx3.2HA, Sox9V5 or Nkx3.2HA+Sox9V5. Immunocytochemistry and qRT-PCR analyses were performed. For all qRT-PCR, GAPDH was used for normalization. (A) Immunocytochemistry analysis of collagen II protein expression in satellite cells. Virus staining images were overlaid with collagen II images, and DAPI stains cell nuclei. (B) Western Blot analysis of collagen II protein expression in satellite cells. ß-actin, internal control. (C) qRT-PCR analysis of collagen II mRNA and (D) Aggrecan mRNA expression. (E) Glycoaminoglycan (GAG) assay. “*” denotes p<0.05 in statistical analysis.

As the differentiation of muscle satellite cells into chondrocytes involves not only the repression of muscle cell fate but also the initiation of chondrogenesis, we next examined the roles of Nkx3.2 and Sox9 in the induction of cartilage genes in muscle satellite cells. We tested if either of these factors could induce chondrogenesis by infecting the satellite cells with viruses expressing Sox9 and/or Nkx3.2. Immunostaining results indicated that either Nkx3.2 or Sox9 infection alone could induce cartilage marker collagen II expression, and that the combination of these two factors had an additive effect, showing a more intense collagen II staining upon Nkx3.2 and Sox9 co-infection (Figure 6A). The protein expression of collagen II was further confirmed by Western blot analysis (Figure 6B). Additionally, qRT-PCR analysis demonstrated that collagen II expression was also elevated at the mRNA level (Figure 6C). To ascertain whether Nkx3.2 and Sox9-infected cells can maintain their chondrocyte phenotype *in vivo*, we implanted the cells into the mouse bone. Under established protocol, mouse humeri were cultured on the chicken chorioallantoic membrane (CAM), which provides nutrient and blood supply to the cultured tissues [31], [32], [33]. We found that Nkx3.2 and Sox9-infected cells maintained collagen II expression (Figure S8).

Interestingly, while Sox9 alone could induce aggrecan expression, Nkx3.2 alone could not (Figure 6C), even in longer-term cultures (Figure S9). However, when combined with Sox9, Nkx3.2 led to a synergistic induction of aggrecan expression (Figure 6D). Thus it is possible that Nkx3.2 and Sox9 regulate the expression of these two cartilage matrix components differently. Finally, we performed a glycosaminoglycan (GAG) assay. This is an established functional assay for differentiated chondrocytes, as cartilage matrix proteoglycans, aggrecan in particular, bind to GAGs (such as heparin sulfate), whose negative charges allow cartilage to resist compression [53], [54], [55]. We found that total GAG content in the satellite cells was significantly induced by Sox9 as well as Nkx3.2/Sox9 co-infection, which is consistent with the qRT-PCR analysis for aggrecan (Figure 6E). Together, these data suggest that Nkx3.2 and Sox9 promote chondrogenic differentiation of muscle satellite cells.

### A reverse function mutant of Nkx3.2 blocks the ability of Sox9 to induce chondrogenesis and inhibit myogenesis in muscle satellite cells

**Figure 7:**
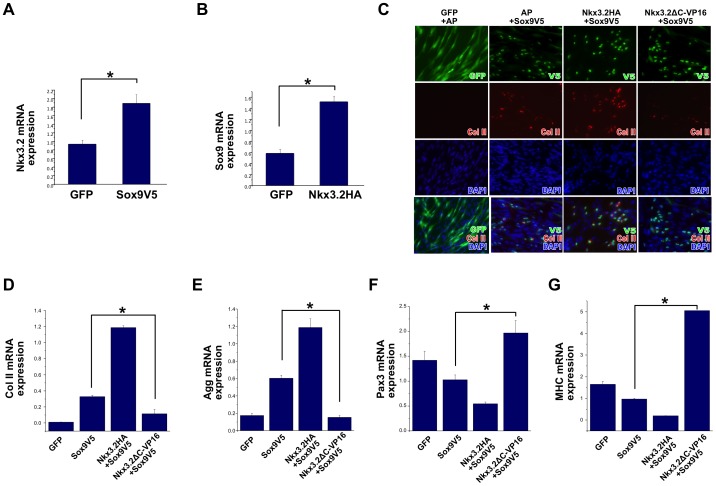
Nkx3.2 is required for Sox9 to activate a cartilage program and to inhibit the muscle program in muscle satellite cells. For all qRT-PCR, GAPDH was used for normalization. (A) qRT-PCR analysis of Nkx3.2 expression in satellite cells infected with control RCAS-GFP virus or RCAS-Sox9V5 virus. (B) qRT-PCR analysis of Sox9 expression in satellite cells infected with control RCAS-GFP virus or RCAS-Nkx3.2HA virus. (C)–(G) Muscle satellite cells were co-infected with the following combination of viruses: RCAS-A-GFP+RCAS-B-AP (alkaline phosphatase); RCAS-A-AP+RCAS-B-Sox9V5; RCAS-A-Nkx3.2HA+RCAS-B-Sox9V5; RCAS-A-Nkx3.2-ΔC-VP16+RCAS-B-Sox9V5. These infected cells were subject to immunocytochemistry analysis and qRT-PCR. (C) Immunocytochemistry analysis of Collagen II protein expression in satellite cells. Virus staining images were overlaid with Collagen II images, and DAPI stains cell nuclei. (D) Collagen II mRNA expression. (E). Aggrecan mRNA expression. (F). Pax3 mRNA expression. (G). MHC mRNA expression. In A–B, “*” denotes p<0.05 in statistical analysis. In D–G, “*” denotes statistically significant differences (p<0.05) between RCAS-A-AP+RCAS-B-Sox9V5 and RCAS-A-Nkx3.2-ΔC-VP16+RCAS-B-Sox9V5 co-infected samples.

Since both Nkx3.2 and Sox9 can promote the chondrogenic differentiation of satellite cells, we investigated the relationship between these factors. We found that indeed, Nkx3.2 and Sox9 induced each other's expression in satellite cells (Figure 7A and 7B). Sox9 is known to strongly induce chondrogenesis by directly activating the promoters of cartilage matrix genes [45], [56], [57]. Our data support this notion and further demonstrate that Sox9 has a weak activity in inhibiting myogenesis. Because Nkx3.2 exerts a stronger inhibitory activity on Pax3, the myogenic factor that can inhibit chondrogenesis, we asked whether the activity of Sox9 on chondrogenesis and myogenesis in satellite cells may be attributed to the induction of Nkx3.2. Therefore, we made use of the reverse function mutant of Nkx3.2 (Nkx3.2-ΔC-VP16), to evaluate whether this mutant, acting in a dominant-negative manner, would compromise the ability of Sox9 [27].

Strikingly, we found that while Sox9-treated satellite cells dramatically upregulated collagen II expression, Nkx3.2ΔC-VP16 co-infection with Sox9 led to a dramatic reduction in this cartilage matrix protein (Figure 7C). This is further confirmed by our RT-PCR analysis that showed diminished expression of collagen II mRNA as well as aggrecan mRNA upon Nkx3.2ΔC-VP16 and Sox9 co-infection (Figure 7D and 7E). We next asked whether Nkx3.2 is required for the weak inhibitory activity of Sox9 on muscle gene expression. Indeed, we found that co-infection of Nkx3.2ΔC-VP16 with Sox9 completely abolished the ability of Sox9 to inhibit Pax3 and MHC expression (Figure 7F and 7G). This suggests that Sox9 is unable to induce chondrogenesis in the absence of the activity of Nkx3.2, and that Sox9 may inhibit the myogenic program in the satellite cells through the induction of Nkx3.2.

### Nkx3.2 and Sox9 are induced in the muscle progenitor cells that contribute to cartilage formation and fracture repair in an in vivo mouse model of bone fracture healing

**Figure 8:**
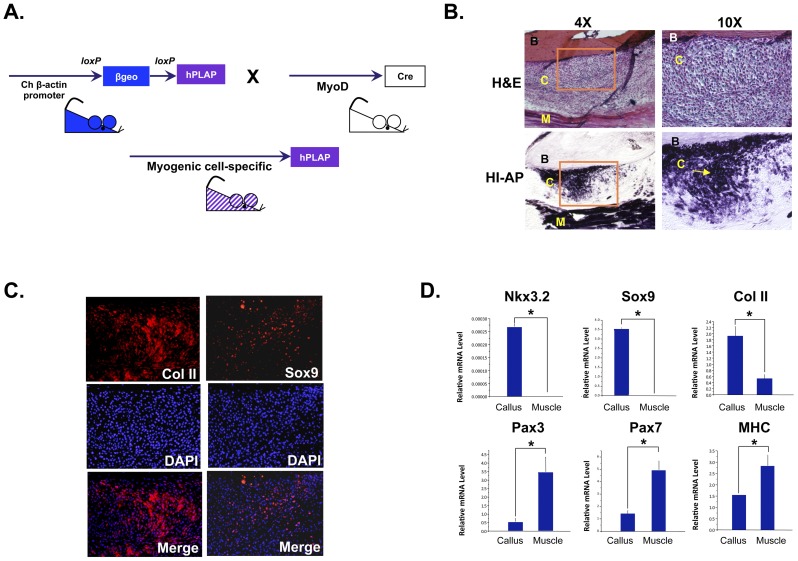
Nkx3.2 and Sox9 are induced in the muscle progenitor cells that contribute to cartilage formation in an *in vivo* mouse model of fracture healing. (A) Schematic diagram illustrating the generation of the transgenic mice in which MyoD+ lineage cells are labeled with heat-resistant alkaline phosphatase (hPLAP). (B) H&E histological analysis showing the fracture callus site 1 week post-fracture. Muscle progenitor cells were identified by assaying for heat-resistant alkaline phosphatase (arrow). Left panels, low magnification (4×); right panels, high magnification of the boxed areas from the left panels (10×). B, bone. C, callus, M, muscle. (C) Immunohistochemistry analyses of collagen II and Sox9 expression in the fracture callus. DAPI staining images were overlaid with collagen II and Sox9 staining images. (D) Laser Capture Microscopy (LCM) analysis of muscle progenitor cells in the fracture callus and in the neighboring muscle. qRT-PCR analyses were performed for the following genes: Nkx3.2, Sox9, collagen II, Pax3, Pax7 and MHC. For all qRT-PCR, 18S RNA was used for normalization. “*” denotes p<0.05 in statistical analysis.

To establish the *in vivo* significance of Nkx3.2 and Sox9 in the chondrogenic differentiation of muscle satellite cells, we evaluated the expression of these factors in the myogenic progenitor cells that give rise to chondrocytes during the process of bone fracture healing. The MyoD-cre: Z/AP mouse was generated by crossing two transgenic lines as described in Materials and Methods (Figure 8A). The MyoD-driven Cre mouse line allows Cre to be expressed in all muscle progenitor cells, including satellite cells [4]. Upon Cre-mediated recombination, the Z/AP line permanently expresses the human placental alkaline phosphatase (hPLAP) reporter gene in affected cells (Figure 8A). The heat-stable property of hPLAP enabled us to distinguish the expression of this reporter from that of endogenous alkaline phosphatase, which is abundantly expressed in bone cells [9], [13]. Therefore, in this MyoD-Cre+∶Z/AP+ mouse, all satellite cells and their progenitors express heat-stable alkaline phosphatase, which is marked by purple staining from enzymatic reactions (Figure 8A). When the MyoD-Cre+∶Z/AP+ mouse was subject to open tibial midshaft fractures, muscle progenitor cells or their descendants were abundantly seen in the fracture callus region (Figure 8B). This callus region is known to undergo cartilage and bone differentiation during the process of fracture healing [58], [59]. In addition to contributing to the fracture callus, it is evident that hPLAP+ cells also marked the muscle next to the bone, and did not mark any of the endogenous osteocytes within the bone (Figure 8B), confirming the validity of this method.

To evaluate whether Sox9 and Nkx3.2 were induced in the muscle progenitor cells, we performed immunohistochemistry (IHC) on sections of the fracture region (Figure 8C). We found that Sox9 was strongly expressed in the cells in the fracture callus, which correlated with the induction of cartilage marker collagen II expression (Figure 8C). Due to the lack of a suitable antibody against Nkx3.2, we were unable to perform IHC to evaluate Nkx3.2 protein expression. Thus, to evaluate Nkx3.2 expression and to confirm that Nkx3.2 and Sox9 are indeed induced in the muscle progenitor cells that give rise to chondrocytes in the fracture healing process, we performed laser capture microscopy (LCM). We captured hPLAP+ muscle progenitor cells from the fracture callus region of frozen tissue sections using alkaline phosphatase (hPLAP) staining on slides of serial sections as a reference. We then compared the expression of muscle and cartilage markers in these muscle progenitor cells from the fracture callus to that of neighboring muscle tissue (Figure 8D). We found that muscle progenitor cells in the fracture callus strongly expressed Nkx3.2 and Sox9, as well as collagen II, as compared with cells in the muscle (Figure 8D). In the meantime, these cells downregulated the expression of muscle markers Pax3, Pax7 and MHC (Figure 8D). These data thus suggest that Nkx3.2 and Sox9 are indeed expressed in the muscle progenitor cells that contribute to cartilage formation during fracture healing, which is consistent with the notion that they promote chondrogenic differentiation of satellite cells.

## Discussion

In this work we show that the muscle satellite cells that normally undergo myogenesis can be converted to express cartilage matrix proteins *in vitro* upon treatment with chondrogenic medium containing TGFß or BMP2. In the meantime, the myogenic program is repressed, suggesting that muscle satellite cells have undergone chondrogenic differentiation. Furthermore, we demonstrate that muscle-determining factor Pax3 strongly inhibits chondrogenesis in these cells, while Nkx3.2 and Sox9 act downstream of TGFß or BMP to promote this cell fate transition. Importantly, our data suggest that Nkx3.2 is required for Sox9 to inhibit myogenesis and induce chondrogenesis. Finally, in the muscle progenitor cells that give rise to chondrocytes in an *in vivo* model of fracture healing, Nkx3.2 and Sox9 are significantly upregulated while Pax3 is significantly downregulated. These events correlated with the induction of cartilage matrix protein collagen II in lineage-traced muscle progenitor cells, suggesting that the balance of Pax3, Nkx3.2 and Sox9 may play an important role in the cell fate switch from muscle to cartilage, which may be important for fracture healing.

Multiple other progenitor cell populations are present in the muscle that can be instructed to adopt alternative cell fates. While muscle satellite cells reside underneath the basal lamina of the myocytes [3], a fibrocyte or adipocyte population (FAP) has been identified in the interstitial spaces of the muscle fibers [39], [40]. These cells do not express muscle satellite cell marker Pax7 or SM/C-2.6, but are positive for Sca1, Tie-2 and PDGFR-1a [39], [40]. While this FAP population can give rise to adipocytes, it cannot be induced to become myogenic or chondrogenic. On the other hand, a Sca-1-negative (also lin-negative) population (i.e. the double-negative (DN) population) in the muscle was found to be capable of differentiating into cartilage and bone, but incapable of differentiating into myocytes [39]. Another progenitor population is the muscle-derived stem cell population (MDSC) [60], [61]. Unlike muscle satellite cells or the FAP population, these MDSCs reside within the basal lamina [61]. MDSCs are also positive for Sca-1 and negative for Pax7, and have the ability to give rise to muscle, cartilage or bone cells [61]. As our cells are positive for Pax3, Pax7, CD34 and M-cadherin, and negative for CD45 and Sca-1, we believe that the cells used in our experiments do not belong to the above populations. However, it is plausible that similar molecular mechanisms are involved in the chondrogenic differentiation of FAP or MDSC cells.

It has been established by the Goldhamer laboratory that the MyoD(+) progenitors permanently label muscle satellite cells as well as their derivatives in the mature muscle fibers [4]. While Goldhamer and colleagues showed that these muscle progenitor cells did not give rise to non-myogenic adipocytes [62], it was not clear whether they have the capacity to adopt a chondrogenic or osteogenic fate. In our study, we evaluated the expression of Nkx3.2, Sox9 and Pax3 in the muscle progenitors that contribute to cartilage formation during bone healing during fracture repair. We demonstrate that muscle progenitor cells adopt a cartilage cell fate upon chondrogenic stimulation *in vitro*, as well as during open fracture healing *in vivo*. However, we did not distinguish which specific subpopulations of satellite cells are more likely to undergo chondrogenesis [2]. *In vitro* expansion of stem-like cells often results in a loss of “stemness” as well as proliferative capacity [63]. It was reported that muscle progenitor cells have decreased proliferation and myogenic potential upon passaging, which is correlated with increased senescence [64], [65]. Interestingly, while muscle satellite cells have an increased tendency to adopt a fibrocyte fate during aging [66], our data showed that they have a reduced capacity to become chondrogenic when passaged. It is possible that cellular senescence contributes to our observations. It is also possible that prolonged monolayer culture of these cells could potentially hinder their ability to form the 3D condensations required for chondrogenesis [67]. We do not know whether these muscle progenitor cells have undergone de-differentiation/re-differentiation or *bona fide* transdifferentiation in our *in vitro* cell culture or *in vivo* fracture healing models. While there was a significant amount of Pax3 and Pax7 protein expression at the beginning of culturing, Pax3 and Pax7 became gradually diminished upon Nkx3.2 and Sox9 viral infection, concurrently with the induction of cartilage genes, which should be consistent with a transdifferentiation process. Msx1 is correlated with muscle cell dedifferentiation [68], [69]. However, msx1 is also highly expressed in chondrocytes and is induced by BMP/TGFß signaling. Thus, although we observed a significant induction of msx1 expression upon chondrogenic differentiation in the satellite cells (data not shown), it does not indicate whether the satellite cells have undergone dedifferentiation. Regardless, our data support that muscle progenitor cells that normally would undergo myogenesis, can be redirected to adopt a cartilage cell fate *in vitro* and *in vivo*.

In this study, we have evaluated cartilage gene expression in the muscle progenitor cells that contribute to fracture healing [70]. However, other cell types located in the vicinity of bone may also participate in cartilage and bone formation. Elegant grafting experiments using LacZ-positive donor mice and Lac-Z-negative recipients revealed that cells from the perichondrium, the fibrous covering of the bone, differentiate into chondrocytes and osteocytes during fracture repair [71]. Cells associated with blood vessels, such as pericytes, have also been shown to have the ability to differentiate into chondrocytes [72]. Cells that are positive for Tie-2, an endothelial cell marker, while not yet shown to be recruited to the fracture callus, are known to contribute to cartilage and bone formation during heterotopic ossification [73], [74]. Because of the diverse cell types that participate in cartilage formation during fracture healing, it is likely that these different types of cells use different signaling mechanisms when undergoing chondrogenic differentiation. It is known that TGFß, BMP, PTH, as well as Wnt signaling are all activated during fracture healing, and downstream molecules such as Smad, prostaglandin, Cox-2 and ß-catenin regulate this process [58], [59], [75], [76], [77], [78], [79], [80], [81], [82], [83], [84]. Our work demonstrates that transcription factors Pax3, Nkx3.2 and Sox9 regulate chondrogenic differentiation of muscle progenitor cells. However, it is unclear whether Nkx3.2 and Sox9 also participate in the chondrogenic differentiation of other cell types, such as perichondrial or endothelial cells, and how these different cell types coordinate their signaling events during fracture healing. The understanding of such signaling processes in different cell types may help to accelerate fracture healing.

Pax3, Nkx3.2 and Sox9 are all known to play important roles during development. In embryogenesis, Pax3 is expressed in the dermomyotome of the somite, which gives rise to muscle cell precursors [41]. Pax3 mutant mice exhibit somite truncations with loss of hypaxial dermomyotome, and absence of limb muscle [3]. Our data support the role of Pax3 in promoting myogenesis in muscle satellite cells [85]. Furthermore, our data shows that Pax3 has an additional function of inhibiting chondrogenic differentiation of muscle satellite cells. It was reported that constitutive expression of Pax3 led to increased proliferation and decreased cell size in satellite cells [86]. We found that Pax3-infected cells had a much more elongated appearance as compared to the control cells cultured in the chondrogenic medium, although we could not clearly distinguish the differences in cell shape due to cell condensation that accompanies chondrogenesis. In the double knockout of Pax3 and its paralogue Pax7, significant cell death takes place, leading to the loss of most muscle fibers [3]. In addition, Pax3 and Pax7 double mutant cells were found in the forming rib [3], suggesting that they may have adopted a cartilage fate, a result consistent with our findings [25], [87]. While Pax3 acts as a transcriptional activator to promote myogenesis [88], it also has a transcriptional repressor domain that is important for the development of melanocytes [89], [90], [91], [92], [93]. It will be of interest to determine whether Pax3 inhibits chondrogenesis by acting as a transcriptional repressor or activator in the satellite cells. It will be also of interest to investigate whether other myogenic factors play inhibitory roles in chondrogenic differentiation.

We also discovered a novel function for Sox9 in this study. Sox9 is the master regulator of chondrogenesis, as no cartilage formation takes place in the absence of Sox9 [46]. Sox9 acts as a transcriptional activator in chondrogenic precursor cells by binding to the promoters of cartilage-specific matrix genes collagen II and aggrecan [45], [56], [57]. We found that Sox9 strongly induced collagen II and aggrecan expression, as well as glycosaminoglycan (GAG) level in the muscle satellite cells, which normally are non-chondrogenic precursors, consistent with its activity in the somite [25]. In the meantime, Sox9 also significantly, although weakly, inhibited the expression of early muscle lineage marker Pax3 and Pax7, as well as myosin heavy chain (MHC). It has been reported that Sox9 is expressed in the satellite cells, and has the ability to inhibit α-sarcoglycan expression in the C2C12 myoblast cell line [94] and the myogenin promoter in 10T1/2 cells [95]. Our data are consistent with these reports. While Sox9 may be expressed in satellite cells, it is apparent from our work and others that Sox9 is much more strongly expressed in chondrocytes, and that ectopic expression of Sox9 leads to chondrogenic differentiation and maintenance of the chondrocyte phenotype [96], [97].

Our data suggest that Nkx3.2 plays a central role in the chondrogenic differentiation of satellite cells, and that its activity is required for Sox9 to promote chondrogenesis and inhibit myogenesis. Like Sox9, Nkx3.2 is expressed in the cartilage precursors in the embryo, and promotes cartilage cell fate in the somites [25], [26]. Nkx3.2 null mice exhibit reduced cartilage formation including a downregulation of Sox9 expression [48], [98], [99]. Inactivating mutations of Nkx3.2 in human lead to spondylo-megaepiphyseal-metaphyseal dysplasia (SMMD), a disease that causes abnormalities of the vertebral bodies, limbs and joints [100]. Here we show that Nkx3.2 is activated in the muscle satellite cells during chondrogenic differentiation *in vitro* as well as in the adult fracture healing process *in vivo*, suggesting that Nkx3.2 may also be involved in a cell fate determination process at a stage later than early embryogenesis. Furthermore, we show that Nkx3.2 acts as a transcriptional repressor to inhibit Pax3 promoter activity. While there are consensus Nkx3.2 binding sites on the Pax3 promoter, we have not determined whether Nkx3.2 binds to the Pax3 promoter [28]. Interestingly, Nkx3.2 has also been shown to act as a repressor to inhibit osteogenic determining factor Runx2, suggesting that Nkx3.2 may be used to inhibit other non-cartilage cell fates [101]. We have also uncovered a pivotal role for Nkx3.2 in the induction of chondrogenic genes. We found that without the repressing activity of Nkx3.2, Sox9, despite its ability to bind to collagen II and aggrecan promoters, was unable to activate those genes or inhibit myogenesis. Additionally, Nkx3.2 potentiates the ability of Sox9 to induce aggrecan expression, which may be due to its repression of chondrogenic inhibitor Pax3. This data is consistent with the time course experiment (Figure S4), which indicated that the high level expression of collagen II and aggrecan is clearly correlated with the induction of Nkx3.2, as Sox9 expression is reduced at later stages of chondrogenesis. In all, our findings indicate that the intricate balance of Pax3, Nkx3.2 and Sox9 controls the determination of cartilage and muscle cell fate in muscle satellite cells, and may play important roles in regulating the process of fracture healing. As these factors are also involved in early embryonic cell fate determination, our work supports the notion that healing recapitulates development. Understanding these signaling events may eventually allow us to harness these mechanisms of chondrogenic differentiation to enhance fracture healing.

## Supporting Information

Figure S1 **Quantitative characterization of muscle satellite cells.** **A.** Percentage of cells positive for Pax3 (96%), Pax7 (95%), M-cadherin (97%), CD34 (98%), Myf5 (33%), Desmin (1%), CD45 (1%), Sca-1 (0%). **B.** qRT-PCR analysis showing satellite cells express high levels of Pax3 and Pax7 mRNA as compared to chick fibroblasts (CEF). **C.** Isolated satellite cells were able to differentiate into myoblasts and myocytes. Immunocytochemistry analysis shows that after 8 days of culture, nearly all cells express myoblast and myocyte markers Desmin. Some cells have already fused and become MHC-positive, and expressed a much higher level of Desmin than myoblasts.
(TIF)

Figure S2 **Efficiency of satellite cell chondrogenesis.** Micromass cultures were dissociated in order to investigate the percentage of cells that were positive for cartilage marker collagen II by immunocytochemistry. **A.** Immunocytochemistry images showing that cells in the control micromass cultures often showed a more elongated nuclei, and had very little collagen II protein expression. In contrast, cells in the micromass cultured in chondrogenic medium had larger and rounder-shaped nuclei, and the majority of them had significant collagen II staining (arrows). **B.** Quantification of collagen II-positive cells. “*” denotes p<0.05 in statistical analysis.
(TIF)

Figure S3 **Chondrogenic potential of satellite cells.** **A.** Pax3 and Pax7 expression is reduced in satellite cells of a later passage (P2) as compared with freshly isolated cells (P0). **B.** Chondrogenic potential of satellite cells of P0 and P2 passages. When satellite cells were cultured as micromasses in chondrogenic medium, P2 satellite cells showed a much reduced potential to express cartilage marker aggrecan, but did not show any differences in collagen II expression. “*” denotes p<0.05 in statistical analysis.
(TIF)

Figure S4 **A time course of chondrogenic differentiation of muscle satellite cells.** Muscle satellite cells were subject to chondrogenic differentiation in micromass cultures, and their gene expression was assayed at day 2, 5 and 12. **A.** Pax3, Pax7 and MyoD expression was significantly reduced in satellite cells cultured in chondrogenic medium over the course of 12 days. **B.** Nkx3.2 expression was significantly increased in satellite cells cultured in chondrogenic medium over the course of 12 days. However, Sox9 expression became reduced by day 12. Aggrecan expression reached a plateau at day 5. Interestingly, we did not detect any significant increase in collagen II expression until day 12. “*” denotes p<0.05 in statistical analysis, when D5 and D12 expression was compared with that of D2.
(TIF)

Figure S5 **Pax3 infection in muscle satellite cells causes a flattened appearance in micromass cultures.** Muscle satellite cells infected with retrovirus GFP (control) or Pax3 were cultured in chondrogenic media as micromasses for 5 days, then cryosectioned and stained with H&E. Micromass cultures of Pax3-infected cells (B) showed an elongated and flattened appearance as to the spherical appearance of control cultures (A). As cells need to be condensed for chondrogenesis to take place, it is difficult to discern the cell shape within these micromass cultures.
(TIF)

Figure S6 **Evaluation of other lineage markers upon Pax3 infection in muscle satellite cells.** Muscle satellite cells infected with retrovirus GFP (control) or Pax3 were cultured in chondrogenic media as micromasses for 5 days. Pax3 did not significantly alter the expression of adipocyte marker PPARγ, bone marker osteopontin, or fibrocyte marker vimentin.
(TIF)

Figure S7 **Nkx3.2HA and Sox9V5 are expressed at the mRNA level in Nkx3.2HA and Sox9V5-infected cells.** qRT-PCR showing viruses encoding Nkx3.2HA and Sox9V5 led to the expression of Nkx3.2HA and Sox9V5 expression. “*” denotes statistically significant differences (p<0.05) relative to control samples.
(TIF)

Figure S8 **Nkx3.2HA and Sox9V5-infected satellite cells maintain collagen II expression** ***in ovo*** **.** Nkx3.2HA and Sox9V5-infected satellite cells were seeded into a 3D scaffold (silk-derived) and implanted into a mouse bone (humerus, fractured for cell implantation), and allowed to grow for 7 days in vivo on the chicken chorioallantoic membrane (CAM). As mouse cells cannot be infected by avian retroviruses, they can be distinguished from implanted chicken satellite cells. **A.** Image of the mouse bone cultured on top of the CAM inside the egg shell. **B.** Bright field image of the bone. Dotted line highlights the outline of the sectioned bone and implanted construct. Boxed area denotes the location where cells were implanted. **C.** Immunostaining images of chicken satellite cells. Implanted Sox9V5-positive cells expressed collagen II (arrows). Additional collagen II staining may come from host cells.
(TIF)

Figure S9 **Nkx3.2 does not induce aggrecan expression in long-term cultures.** Muscle satellite cells infected with retrovirus GFP (control) or Nkx3.2 were cultured in chondrogenic media as micromasses for 5 or 12 days. The expression levels of aggrecan between GFP and Nkx3.2-infected samples at both time points were not statistically significant. Nkx3.2 thus may require additional factors to induce aggrecan expression.
(TIF)

Table S1 **RT-PCR primer sequences.**
(PDF)
