# Supplementary figures and images for: Interplay of Nkx3.2, Sox9 and Pax3 Regulates Chondrogenic Differentiation of Muscle Progenitor Cells
Source: PLoS One. 2012 Jul 2;7(7):e39642. doi: 10.1371/journal.pone.0039642 (PMC3388093; doi:10.1371/journal.pone.0039642)

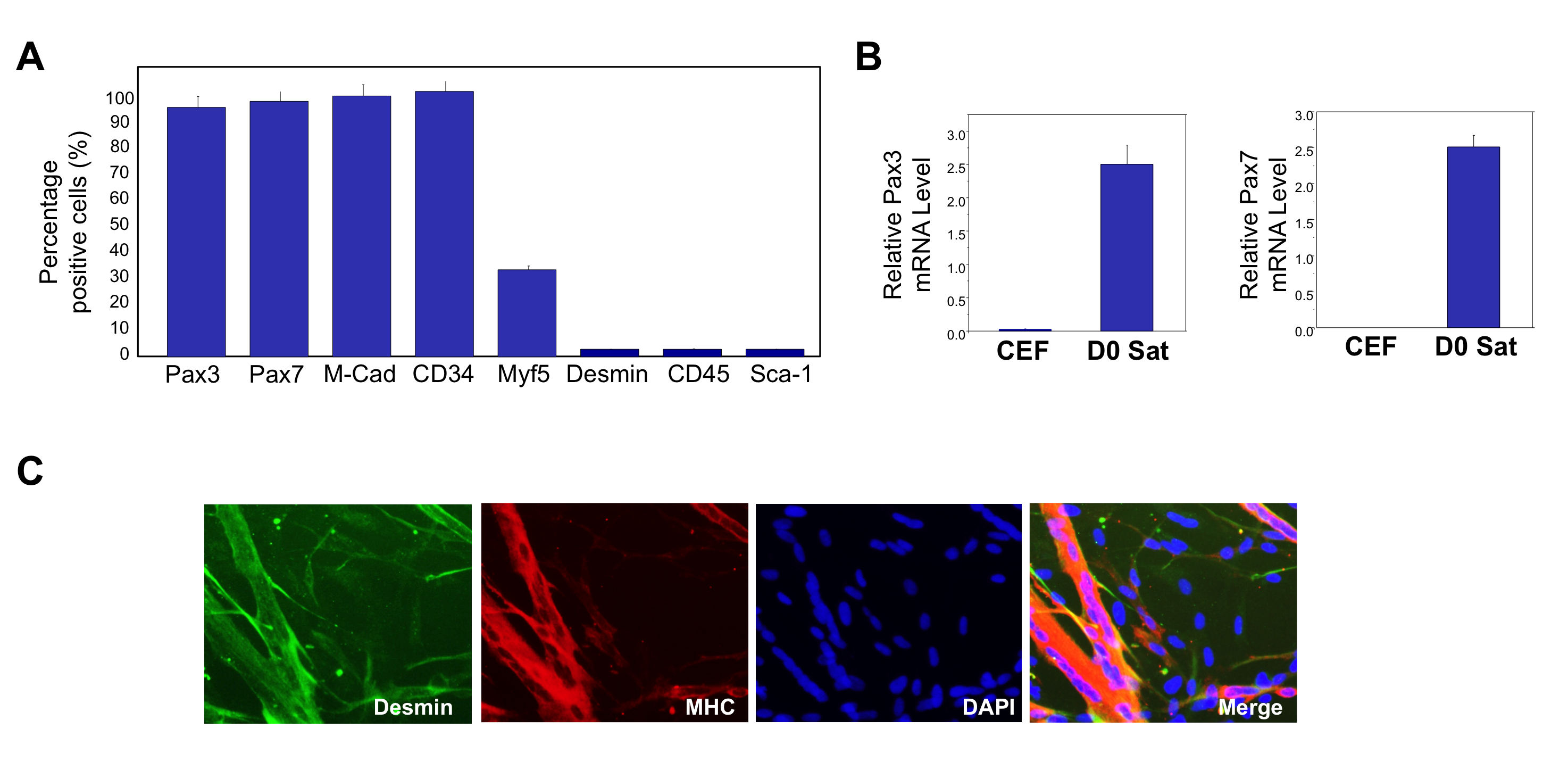

Supplement: Figure S1 — Quantitative characterization of muscle satellite cells. A. Percentage of cells positive for Pax3 (96%), Pax7 (95%), M-cadherin (97%), CD34 (98%), Myf5 (33%), Desmin (1%), CD45 (1%), Sca-1 (0%). B. qRT-PCR analysis showing satellite cells express high levels of Pax3 and Pax7 mRNA as compared to chick fibroblasts (CEF). C. Isolated satellite cells were able to differentiate into myoblasts and myocytes. Immunocytochemistry analysis shows that after 8 days of culture, nearly all cells express myoblast and myocyte markers Desmin. Some cells have already fused and become MHC-positive, and expressed a much higher level of Desmin than myoblasts. (TIF) [file pone.0039642.s001.tif]

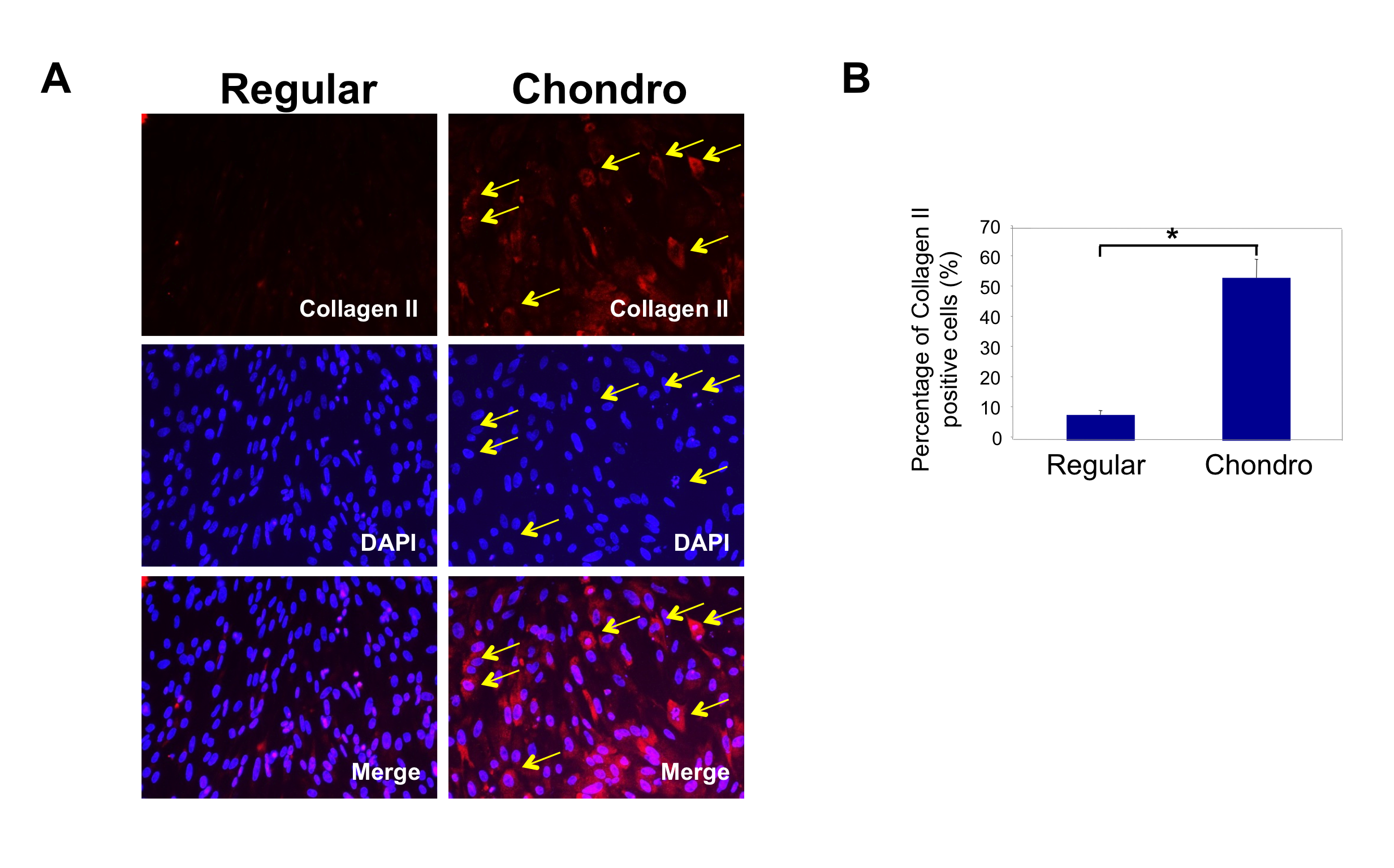

Supplement: Figure S2 — Efficiency of satellite cell chondrogenesis. Micromass cultures were dissociated in order to investigate the percentage of cells that were positive for cartilage marker collagen II by immunocytochemistry. A. Immunocytochemistry images showing that cells in the control micromass cultures often showed a more elongated nuclei, and had very little collagen II protein expression. In contrast, cells in the micromass cultured in chondrogenic medium had larger and rounder-shaped nuclei, and the majority of them had significant collagen II staining (arrows). B. Quantification of collagen II-positive cells. “*” denotes p<0.05 in statistical analysis. (TIF) [file pone.0039642.s002.tif]

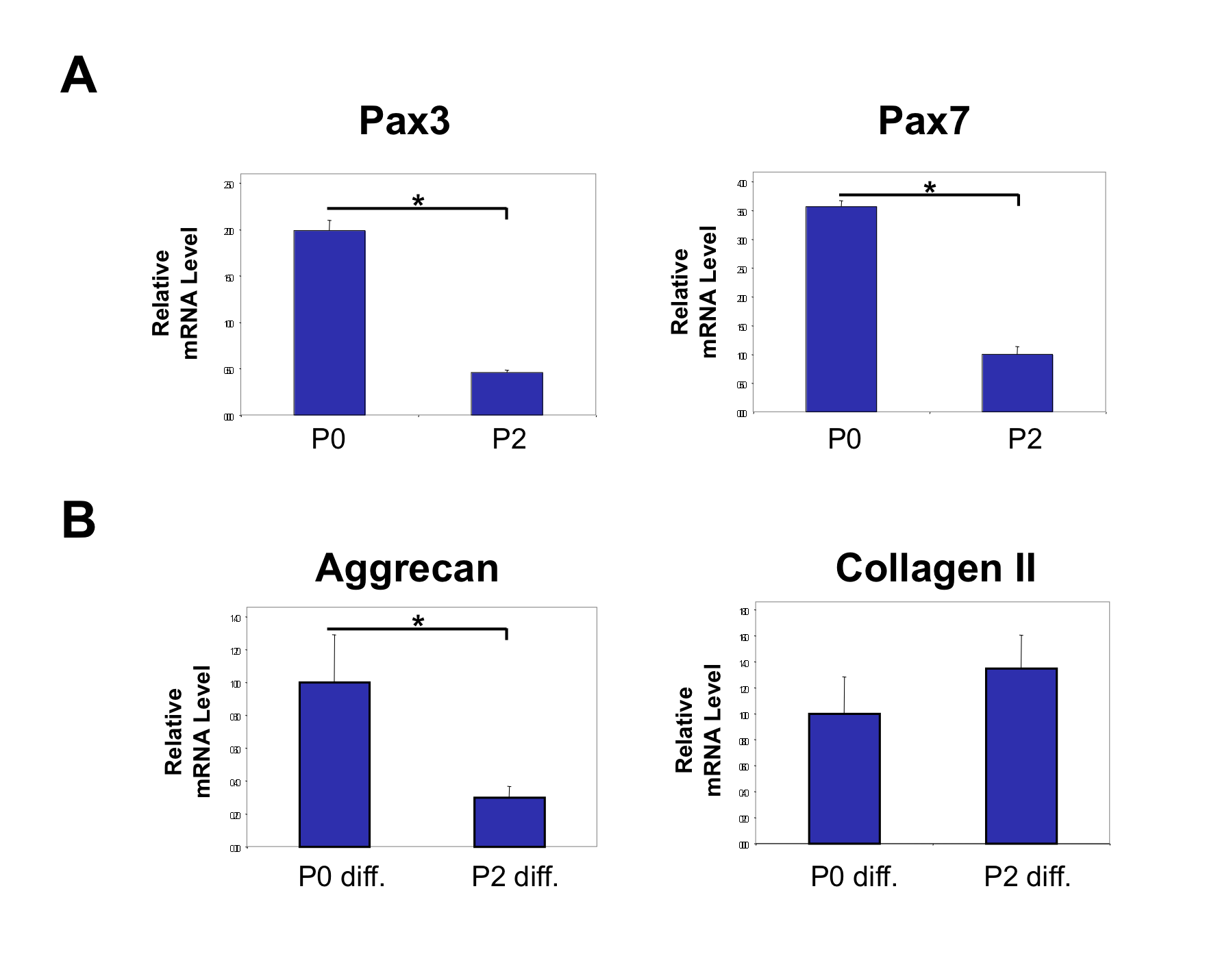

Supplement: Figure S3 — Chondrogenic potential of satellite cells. A. Pax3 and Pax7 expression is reduced in satellite cells of a later passage (P2) as compared with freshly isolated cells (P0). B. Chondrogenic potential of satellite cells of P0 and P2 passages. When satellite cells were cultured as micromasses in chondrogenic medium, P2 satellite cells showed a much reduced potential to express cartilage marker aggrecan, but did not show any differences in collagen II expression. “*” denotes p<0.05 in statistical analysis. (TIF) [file pone.0039642.s003.tif]

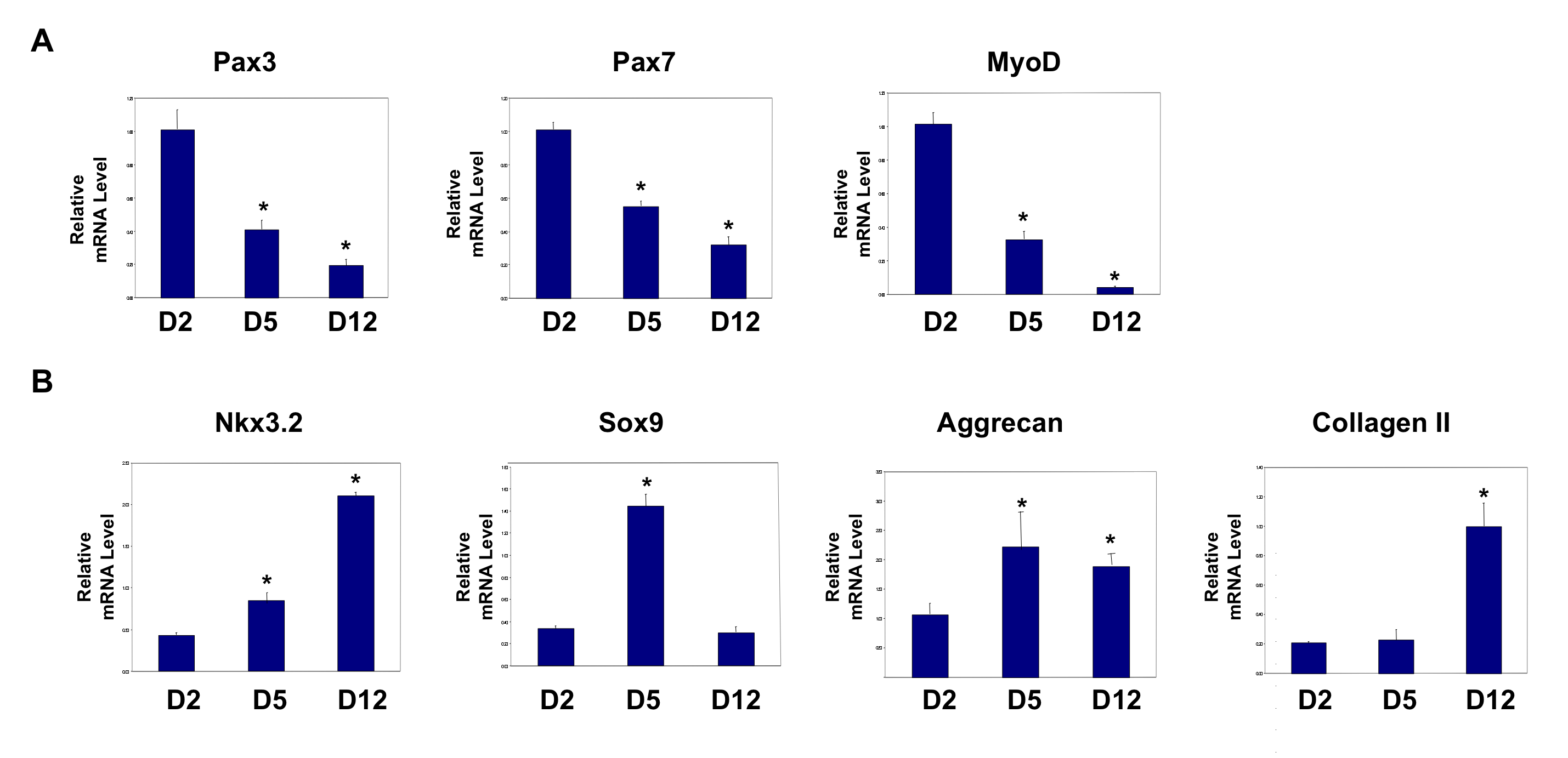

Supplement: Figure S4 — A time course of chondrogenic differentiation of muscle satellite cells. Muscle satellite cells were subject to chondrogenic differentiation in micromass cultures, and their gene expression was assayed at day 2, 5 and 12. A. Pax3, Pax7 and MyoD expression was significantly reduced in satellite cells cultured in chondrogenic medium over the course of 12 days. B. Nkx3.2 expression was significantly increased in satellite cells cultured in chondrogenic medium over the course of 12 days. However, Sox9 expression became reduced by day 12. Aggrecan expression reached a plateau at day 5. Interestingly, we did not detect any significant increase in collagen II expression until day 12. “*” denotes p<0.05 in statistical analysis, when D5 and D12 expression was compared with that of D2. (TIF) [file pone.0039642.s004.tif]

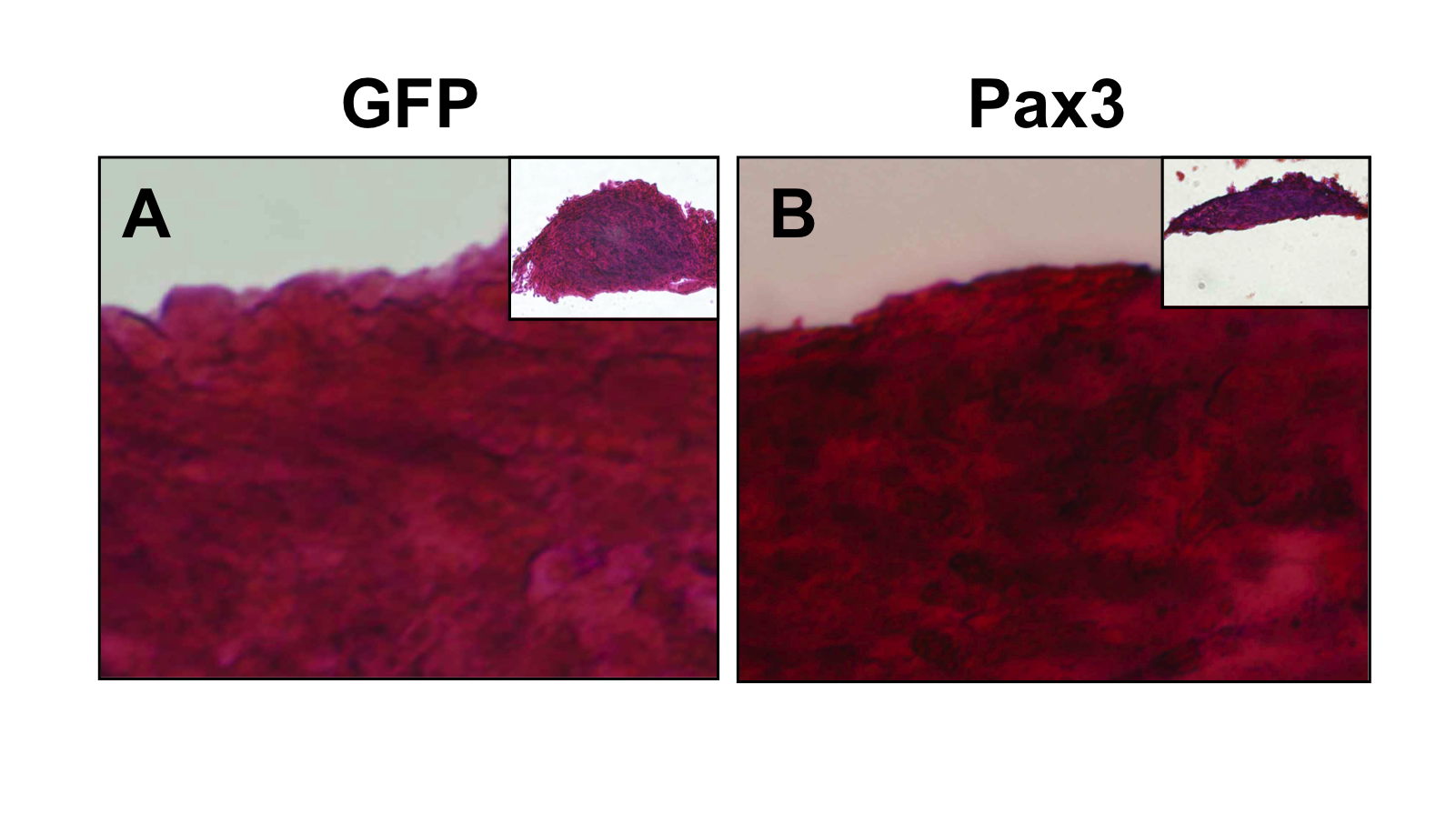

Supplement: Figure S5 — Pax3 infection in muscle satellite cells causes a flattened appearance in micromass cultures. Muscle satellite cells infected with retrovirus GFP (control) or Pax3 were cultured in chondrogenic media as micromasses for 5 days, then cryosectioned and stained with H&E. Micromass cultures of Pax3-infected cells (B) showed an elongated and flattened appearance as to the spherical appearance of control cultures (A). As cells need to be condensed for chondrogenesis to take place, it is difficult to discern the cell shape within these micromass cultures. (TIF) [file pone.0039642.s005.tif]

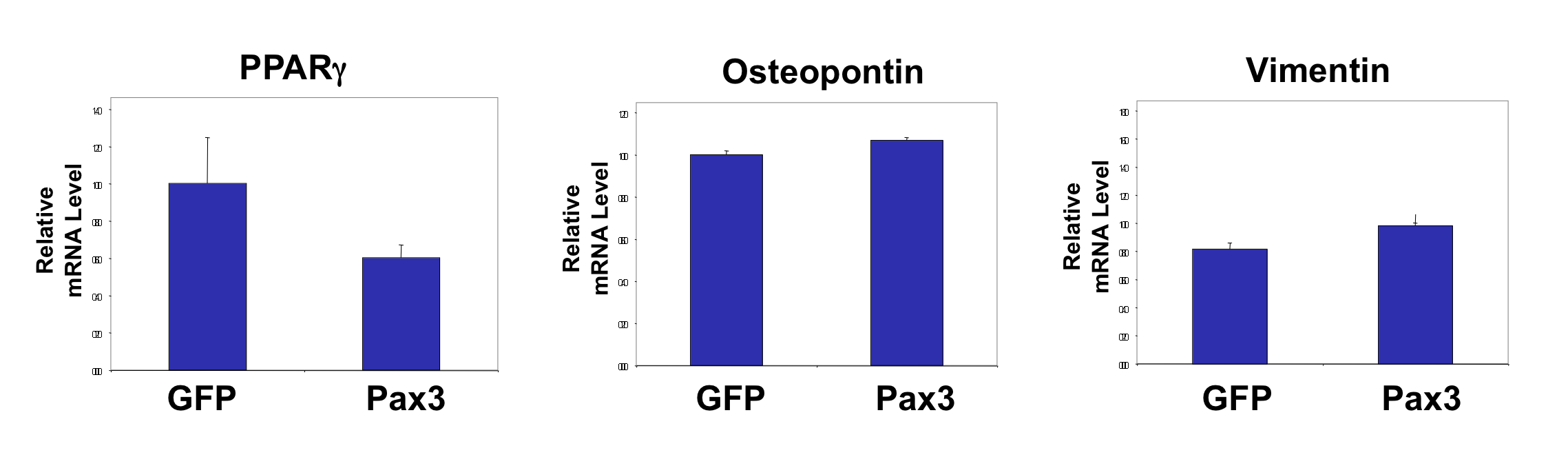

Supplement: Figure S6 — Evaluation of other lineage markers upon Pax3 infection in muscle satellite cells. Muscle satellite cells infected with retrovirus GFP (control) or Pax3 were cultured in chondrogenic media as micromasses for 5 days. Pax3 did not significantly alter the expression of adipocyte marker PPARγ, bone marker osteopontin, or fibrocyte marker vimentin. (TIF) [file pone.0039642.s006.tif]

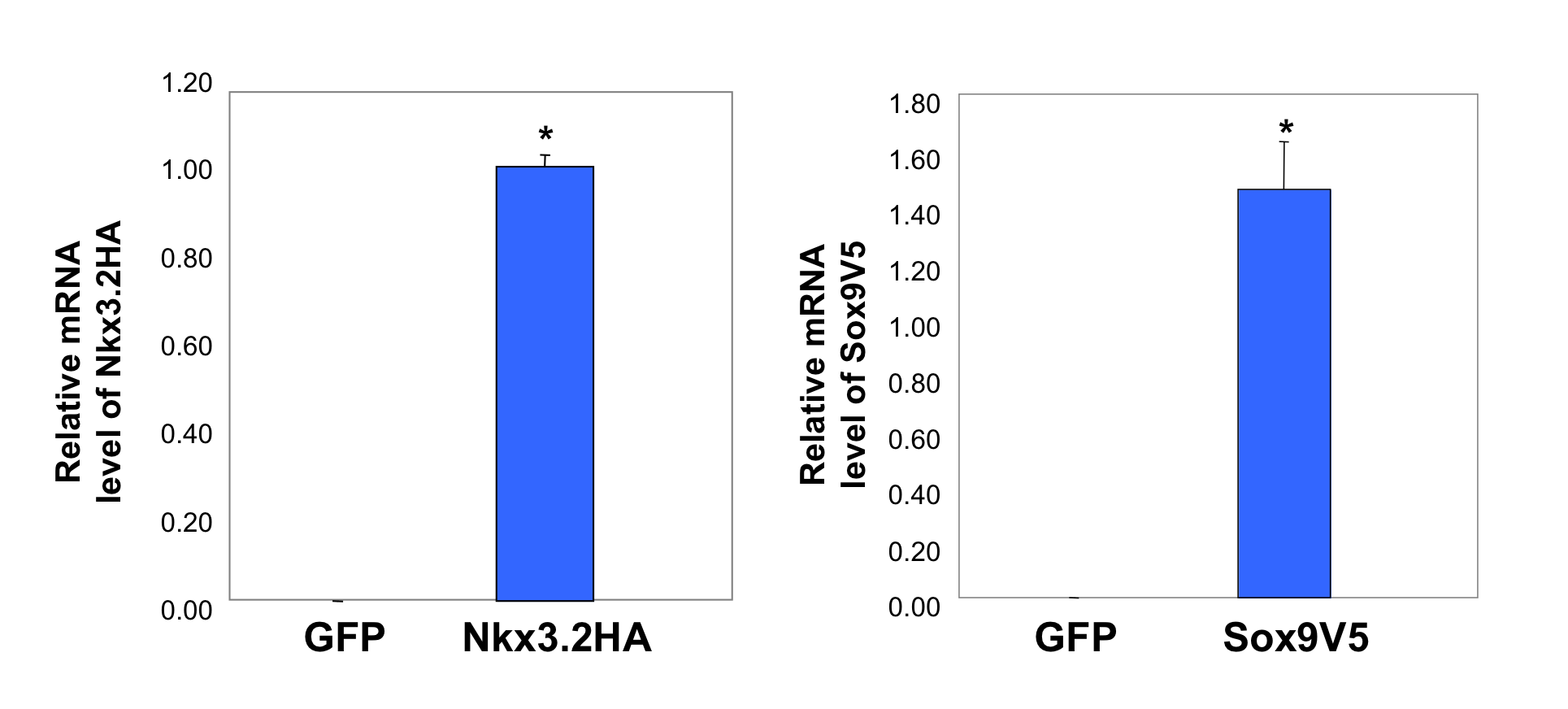

Supplement: Figure S7 — Nkx3.2HA and Sox9V5 are expressed at the mRNA level in Nkx3.2HA and Sox9V5-infected cells. qRT-PCR showing viruses encoding Nkx3.2HA and Sox9V5 led to the expression of Nkx3.2HA and Sox9V5 expression. “*” denotes statistically significant differences (p<0.05) relative to control samples. (TIF) [file pone.0039642.s007.tif]

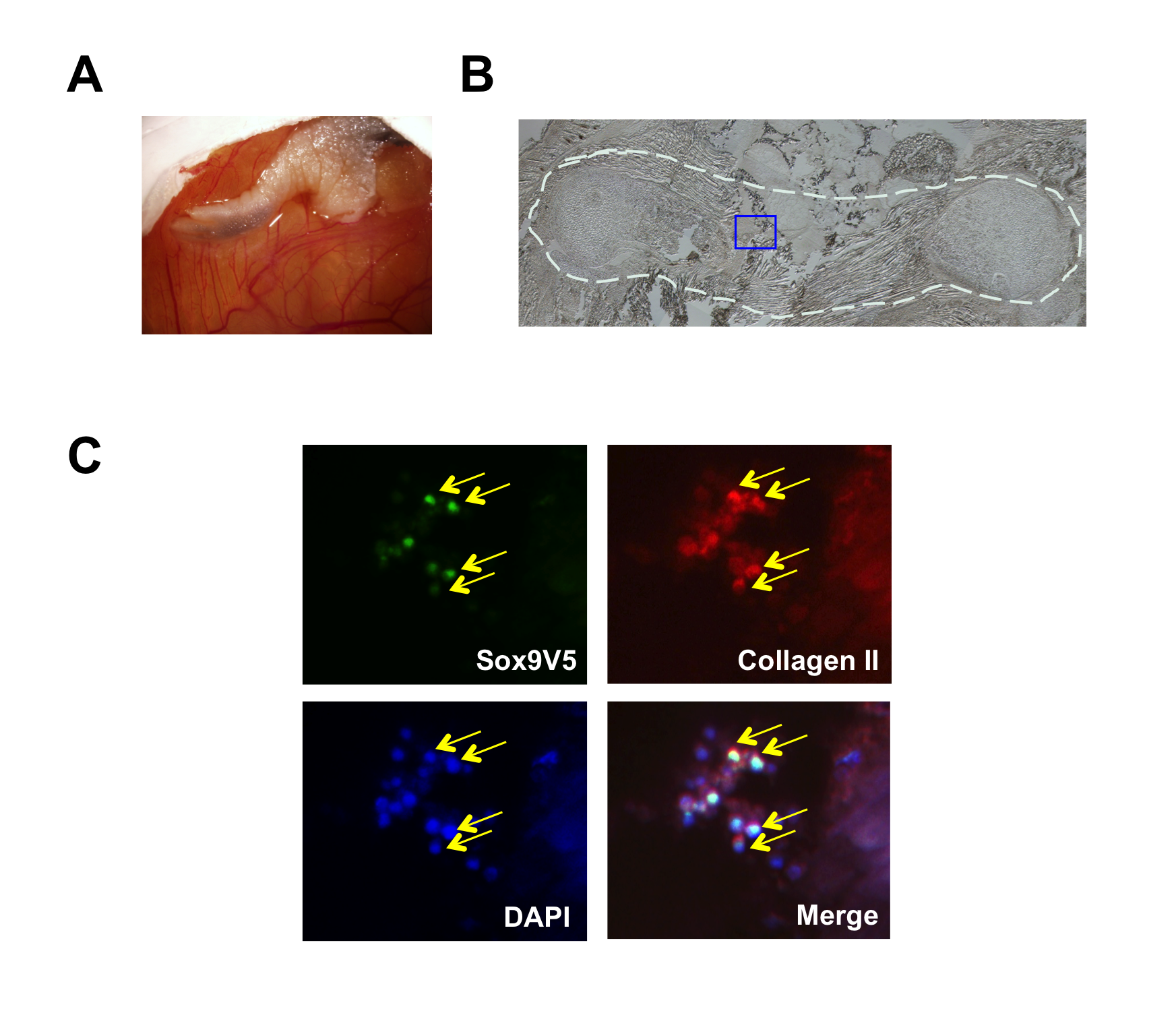

Supplement: Figure S8 — Nkx3.2HA and Sox9V5-infected satellite cells maintain collagen II expression in ovo . Nkx3.2HA and Sox9V5-infected satellite cells were seeded into a 3D scaffold (silk-derived) and implanted into a mouse bone (humerus, fractured for cell implantation), and allowed to grow for 7 days in vivo on the chicken chorioallantoic membrane (CAM). As mouse cells cannot be infected by avian retroviruses, they can be distinguished from implanted chicken satellite cells. A. Image of the mouse bone cultured on top of the CAM inside the egg shell. B. Bright field image of the bone. Dotted line highlights the outline of the sectioned bone and implanted construct. Boxed area denotes the location where cells were implanted. C. Immunostaining images of chicken satellite cells. Implanted Sox9V5-positive cells expressed collagen II (arrows). Additional collagen II staining may come from host cells. (TIF) [file pone.0039642.s008.tif]

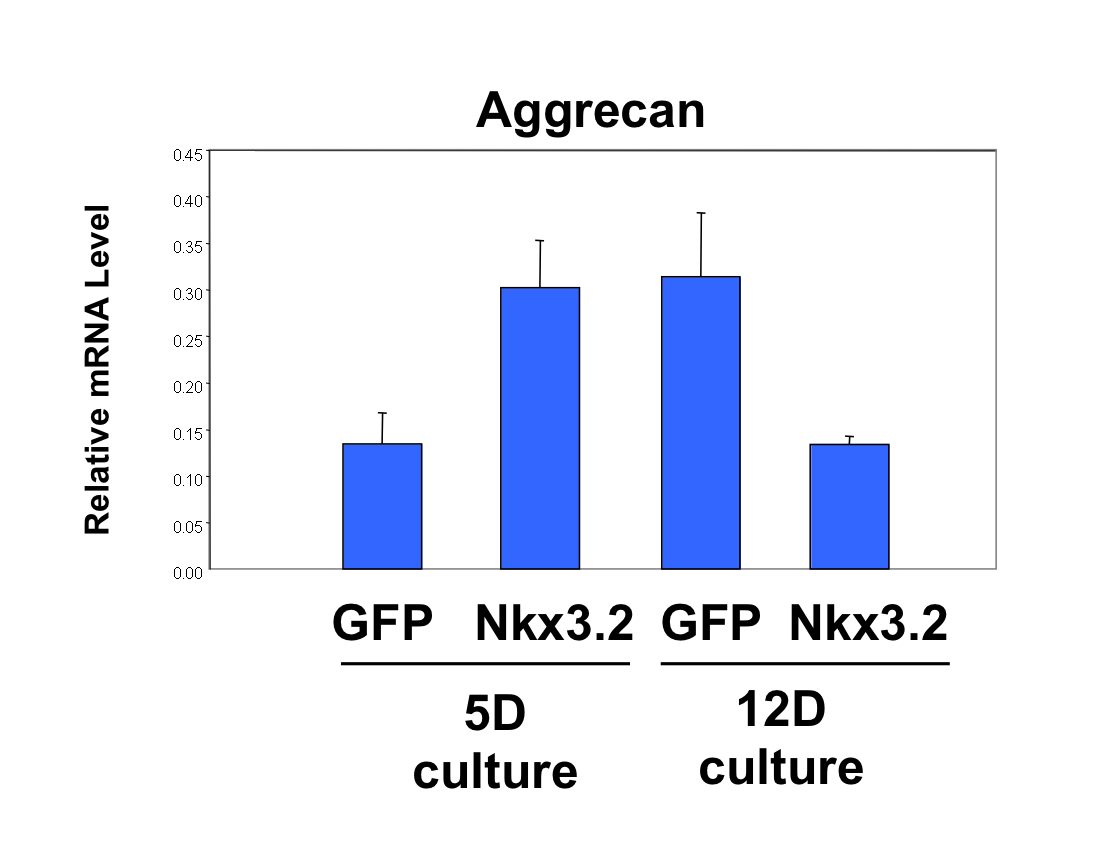

Supplement: Figure S9 — Nkx3.2 does not induce aggrecan expression in long-term cultures. Muscle satellite cells infected with retrovirus GFP (control) or Nkx3.2 were cultured in chondrogenic media as micromasses for 5 or 12 days. The expression levels of aggrecan between GFP and Nkx3.2-infected samples at both time points were not statistically significant. Nkx3.2 thus may require additional factors to induce aggrecan expression. (TIF) [file pone.0039642.s009.tif]
