# Supplementary material for: Interplay of Nkx3.2, Sox9 and Pax3 Regulates Chondrogenic Differentiation of Muscle Progenitor Cells
Source: PLoS One. 2012 Jul 2;7(7):e39642. doi: 10.1371/journal.pone.0039642 (PMC3388093; doi:10.1371/journal.pone.0039642)
Supplement: Table S1 — RT-PCR primer sequences. (PDF) [file pone.0039642.s010.pdf]

Species	Gene & Accession No.	Forward Sequence	Reverse Sequence
Chicken	GAPDH NM_204305.1	5'- CCT GCT GCC TAG GGA AGC -3'	5'- CAG ATC AGT TTC TAT CAG CCT CT -3'
Chicken	Collagen II NM_204426.1	5'- GCA CAA CTT CTG CAC TGA ACG GAT -3'	5'- TCA CAC CTG CCA GAT TGA TTC CCA -3'
Chicken	Aggrecan XM_001232949.1	5'- AGT GAC AAC CCA GTC AGT TGC AGA -3'	5'- AGA AGC GCT CCC ACC AAA GTC TAT -3'
Chicken	Nkx3.2 NM_204137.1	5'- TGC AGC CCT CCT CAC AAG TGT AAT -3'	5'- CGG GCT GCT TAC ACA CAT TCA CAA -3'
Chicken	Sox9 NM_204281.1	5'- GTC TCT GCC GGC TTT ACT TCT TGT -3'	5'- TGC GAG AAA GCG GCA CAG GG -3'
Chicken	Pax3 NM_204269.1	5'- TTC AGG TTT GGT TTA GCA ACC GCC -3'	5'- TAC TGC TTG GAT CAG ACA CGG CTT -3'
Chicken	Pax7 NM_205065.1	5'- AGC CGT GTG CTA CGC ATC AAA TTC -3'	5'- TTC CTC TTC AAA GGC AGG TCT GGT -3'
Chicken	MyoD NM_204214.1	5'- ACG ACA GCA GCT ACT ACA CGG AAT -3'	5'- TCT CCA CAA TGC TTG AGA GGC AGT -3'
Chicken	Myogenin NM_204184.1	5'- TGA AAC CGC CCA AAT CCT TTC CCA -3'	5'- CGA AGA GCA ACT TGG AAA CAG CCA -3'
Chicken	Myosin Heavy Chain (MHC) NM_204228.1	5'- GCA GAA TTT CAG AAG ATG CGC CGT -3'	5'- TGA CTC GTT GCA GGT TGT CGA TCT -3'
Chicken	Nkx3.2HA NM_204137.1 ABW40918.1	5’- AAG GTG GCC GTC AAG GTG CTG GTG -3’	5’- GGC GTA GTC AGG CAC GTC GTA AGG ATA
Chicken	Sox9V5 NM_204281.1 P11207	5’- TGA ACC CCA CGC AGC GCC CCA TGT -3’	5’- ATC CAG TCC CAA CAG TGG GTT CGG GAT - 3’
Chicken	PPARγ NM_001001460.1	5’- TGG ACA AGG ATT CAT GAC ACG GGA	5’- ACA AAC CTG GGC GAT CTC CAC TTA
Chicken	Osteopontin AAA62729	5’- CAT GCC ATT TCT GCC AGC TCT GAA	5’- AAG CCA GGT CAT TCT GTG TCT GCT
Chicken	Vimentin NM_001048076.1	5’- TAA GCC TGA TCT TAC TGC TGC CCT	5’- ATT AGC TTC TTG TTT GGC CTG GCG

Species	Gene & Accession No.	Forward Sequence	Reverse Sequence
Mouse	18S NR_003278.2	5’- TCA ACT TTC GAT GGT AGT CGC CGT -3’	5’- TCC TTG GAT GTG GTA GCC GTT TCT -3’
Mouse	Collagen II NM_001113515.2	5'- ACA TAG GGC CTG TCT GCT TCT TGT -3'	5'- TGA CTG CGG TTG GAA AGT GTT TGG -3'
Mouse	Nkx3.2 NM_007524.3	5'- TCA GAA CCG TCG CTA CAA GAC CAA -3'	5'- CAG CAC CTT TAC GGC CAC TTT CTT -3'
Mouse	Sox9 NM_011448.4	5'- AGG TTT CAG ATG CAG TGA GGA GCA -3'	5'- ACA TAC AGT CCA GGC AGA CCC AAA -3'
Mouse	Pax3 NM_008781.4	5'- TAC CAG CCC ACG TCT ATT CCA CAA -3'	5'- TTT GGT GTA CAG TGC TCG GAG GAA -3'
Mouse	Pax7 NM_011039.2	5’- TTC AAA GGA GGA GAC TGT TGG GCT -3’	5’- TGT GGA GGA GGA TGC ATT TGG TCT -3’
Mouse	Myosin Heavy Chain (MHC) X57377.1	5’- AGG CTT ACA AGC AAA TGG CAA GGG -3’	5’- ACC GCA TGG CAT ACT TAG CAG AGA -3’
